# Computational Repurposing and Experimental Validation of YBX1 Inhibitors in Hepatocellular Carcinoma

**DOI:** 10.3390/biomedicines14030545

**Published:** 2026-02-27

**Authors:** Omar Karkoutly, Veerababu Nagati, Subhash C. Chauhan, Manish Tripathi

**Affiliations:** 1 School of Medicine, University of Texas Rio Grande Valley, Edinburg, TX 78541, USA; omar.karkoutly01@utrgv.edu; 2 Department of Medicine and Oncology, School of Medicine, University of Texas Rio Grande Valley, McAllen, TX 78504, USA; veerababu.nagati@utrgv.edu (V.N.); subhash.chauhan@utrgv.edu (S.C.C.); 3 South Texas Center of Excellence in Cancer Research, School of Medicine, University of Texas Rio Grande Valley, McAllen, TX 78504, USA

**Keywords:** Y-box binding protein 1 (YBX1), multidrug resistance, HTVS, DrugBank, hepatocellular carcinoma

## Abstract

**Background/Objectives:** Hepatocellular carcinoma (HCC) is the most common type of liver cancer worldwide. While early-stage HCC can often be treated with surgical resection, ablation, or liver transplantation, advanced disease typically relies on systemic chemotherapy. Sorafenib is the standard first-line therapy for advanced and unresectable HCC; however, both intrinsic and acquired resistance remain major clinical challenges. The Y-box binding protein-1 (YBX1), a transcription factor implicated in drug resistance across multiple cancers, is highly expressed in HCC and represents a potential therapeutic target. This study aimed to identify novel YBX1 inhibitors using a drug repurposing strategy to overcome sorafenib resistance. **Methods:** A combined in silico and in vitro approach was employed. The cold shock (DNA-binding) domain of YBX1 was modeled, and a comprehensive library of experimental and FDA-approved compounds from the DrugBank database was screened using multi-layered high-throughput virtual screening (HTVS). Candidate compounds with predicted direct interaction with YBX1 were further evaluated through literature review and experimental validation. **Results:** Virtual screening identified 22 potential compounds predicted to interact with YBX1. Further literature review and feasibility assessment narrowed the list to six candidates: malonaldehyde, mercaptoethanol, glycine, para-chlorophenol, methoxyamine, and ethanolamine. For further evaluation, glycine (a food supplement with no toxicity) was selected for detailed functional studies and was shown to inhibit YBX1 and downregulate its target genes. **Conclusions:** These findings support YBX1 as a promising therapeutic target in hepatocellular carcinoma and demonstrate the utility of drug repurposing to rapidly identify candidate inhibitors. Targeting YBX1 may provide a viable strategy for enhancing treatment efficacy and overcoming sorafenib resistance in advanced HCC.

## 1. Introduction

Cancer is the second leading cause of death in the United States overall and the leading cause among people younger than 85 years [1]. Among these, hepatocellular carcinoma (HCC) is the most frequently diagnosed type of liver cancer and ranks second globally in cancer-related deaths [2]. Risk factors that elevate the probability of diagnosing hepatocellular carcinoma (HCC) are all linked to liver cirrhosis, including infections such as hepatitis B or C, non-alcoholic fatty liver disease (NAFLD), non-alcoholic steatohepatitis (NASH), excessive alcohol intake, obesity, and diabetes [3]. HCC incidents have tripled over the past forty years and continue to grow by 2% each year. In the United States alone, it is projected that in 2025, there will be 42,240 new cases diagnosed (28,220 in men and 14,020 in women) and 30,090 deaths [1]. Hispanics, especially Texas-Hispanics, are the second most affected group by liver cancer overall in Texas, as per the American Cancer Society [1]. Anti-HCC chemotherapeutic drugs must reach a specific intracellular concentration to be effective [4]. Multi-drug resistance (MDR) to standard chemotherapeutic treatments is a significant factor in the failure of these cancer therapies [5]. The impaired expression or function of plasma membrane proteins involved in drug transport, including decreased drug uptake or increased drug efflux, is mainly responsible for this drug resistance. The ATP-binding cassette (ABC) protein family plays a key role in MDR because it transports various anticancer agents, including prominent tyrosine kinase inhibitors (TKIs) such as sorafenib, lenvatinib, and regorafenib, which are commonly used as first-line therapies [4]. Among the 51 ABC family proteins, the overexpression of P-glycoprotein 1 (ABCB1 or MDR1), an organic cation pump, stands out clinically because it is strongly associated with a heightened MDR phenotype in many cancer types and human malignancies [6]. Since this transporter is overexpressed in cancers, tumor cells develop resistance to anticancer drugs, such as sorafenib, through chronic exposure and excessive drug efflux from the cell [7]. Furthermore, most current anti-HCC agents, including sorafenib, are hepatotoxic and may be misinterpreted as disease progression or complicate treatment (livertox.nih.gov, 2018).

Given these significant effects, a comprehensive literature review was conducted to identify a potential cancer target that is strongly associated with MDR or MDR-related genes and is highly expressed across various cancer types [8]. This process led to the identification of YBX1. Increased nuclear expression of YBX1 is associated with resistance to many inhibitors [9]. Typically, YBX1 is also found in the cytoplasm, where it participates in the post-transcriptional regulation of mRNA splicing for several genes involved in the epithelial–mesenchymal transition (EMT) [10]. In response to various environmental stimuli, including chronic exposure to anticancer chemotherapeutic drugs, YBX1 translocates to the nucleus and functions as a transcription factor. It binds to the Y-box consensus sequence (5′-CTGATTGG-3′) in the promoter regions of DNA for MDR-related genes, such as ABCB1, MVP/LRP, TOP2A, CD44, CD49, BCL2, and MYC, leading to their dysregulation [7]. Furthermore, higher nuclear expression of YBX1 in cancer cells is closely linked to reduced overall survival (OS) in cancers of the breast, ovary, prostate, liver, stomach, colorectal, and lung. It is also associated with several cancer biomarkers, including ABCB1, MVP/LRP, EGFR, HER2, AR, and CDC6 [7,11]. To further support this, the expression levels of YBX1 were examined in HCC using the TCGA and GTEx databases. The pan-cancer analysis of this information revealed a notable increase in YBX1 expression in tumor tissues, suggesting its potential role as a tumor promoter across various cancer types, including HCC [12,13]. Chronic exposure to chemotherapeutic drugs such as sorafenib and hypoxia-inducible factor 1α and 2α promotes liver fibrosis and contributes to advanced HCC [14,15].

Our preliminary studies, along with those by Chao et al., showed that YBX1 knockdown in HCC cells increased sensitivity to sorafenib compared with the control [16]. These data suggest that although sorafenib may not directly impact YBX1, YBX1 could play a crucial role in drug resistance. Given its involvement in regulating MDR1 expression, cell proliferation, cell cycle, and metastasis across various cancers, and its relatively understudied role in liver cancer, YBX1 represents a highly promising new therapeutic target to make liver cancer cells more sensitive to sorafenib. By utilizing advanced, often overlooked, in silico tools and cutting-edge bioinformatics techniques, such as homology modeling, researchers can analyze protein–drug interactions and discover potential small-molecule inhibitors for cancer targets by assessing their protein–ligand binding affinities. Computational docking and molecular dynamics suggested that fisetin binds to the CSD domain of YBX1 and hinders phosphorylation [17]. In this study, a high-throughput virtual screening (HTVS) method was used, following a series of specified parameters to identify candidate drugs with the strongest binding affinities and lowest toxicity for YBX1. Interestingly, previous research indicates that glycine inhibits angiogenic signaling in HCC and prevents the development of liver tumors [18,19,20]. Our research has demonstrated that glycine suppresses YBX1 and related drug resistance genes in HCC cell line models.

## 2. Materials and Methods

### 2.1. Computational Methods

#### Spotting of YBX1 Protein Binding Domain

The 3D crystal structure of the YBX1 (PDB ID: 6LMR) protein-binding domain was obtained from the RCSB Protein Data Bank and includes the highly conserved cold shock domain, which represents the protein’s DNA-binding domain, and was validated. Once validated, the predicted protein structure is used for downstream protein–drug interaction and docking analyses within Discovery Studio Client (BIOVIA Discovery Studio Visualizer 2020 (v20.1).

### 2.2. Visualization and Validation of the Protein Model

After obtaining the protein model, BIOVIA Discovery Studio Visualizer 2020 (v20.1) was used to visualize it, remove unnecessary molecules (e.g., water and hydrogen atoms), and confirm the absence of structural abnormalities, such as excessive loops or coils. Next, an open access website called PROCHECK (https://www.ebi.ac.uk/thornton-srv/software/PROCHECK/, accessed on 22 February 2026) was used to generate Ramachandran plots by uploading the resulting PDB file to the ProCheck server [21]. The 6LMR model for YBX1 was submitted to the ProCheck server for a Ramachandran plot. The confirmed model then served as a control (wild type), as it contained no known mutations.

### 2.3. Searching for Homologous Binding Regions (Functional Validation of Conserved Binding Domain)

Validation of the conserved binding domain of YBX1 was performed to further justify the findings. After a verified protein model was obtained, a literature search was conducted for other proteins with a similar structure and/or binding domain to YBX1. BLAST (https://blast.ncbi.nlm.nih.gov/Blast.cgi, accessed on 22 February 2026) data were used with the CLUSTAL Omega Multiple-Sequence Alignment tool (https://www.ebi.ac.uk/jdispatcher/msa/clustalo?outfmt=fa, accessed on 22 February 2026) to identify similar consensus sequence sites between these homologous proteins and YBX1 [22].

### 2.4. Drug Compound Retrieval and Preparation

A digital library of drug compounds was retrieved from a central online database in the public domain (go.drugbank.com). The latest release of DrugBank Online (version 5.1.8, released 3 January 2021) contains 14,522 drug entries, including 2683 approved small-molecule drugs, 1464 approved biologics (proteins, peptides, vaccines, and allergenics), 131 nutraceuticals, and over 6654 experimental (discovery-phase) drugs [23,24]. Additionally, 5249 non-redundant protein (i.e., drug target/enzyme/transporter/carrier) sequences are linked to these drug entries.

### 2.5. Multi-Layered High-Throughput Virtual Screening (HTVS) for the Identification of Potential Inhibitors of YBX1

The docking analyses and drug screening of the entire DrugBank library consisted of a series of steps that gradually narrowed a list of potential YBX1 inhibitors. To achieve this, a multi-layered screening process was employed to perform the HTVS [25]. Rigid docking assessed how well drug ligands bind to the fixed shape of the binding pocket. Flexible docking allowed residues in the protein-binding domain to move more naturally. ADMET analysis provided information on the pharmacokinetic properties of these protein–drug interactions.

#### 2.5.1. Rigid Docking Analysis

A preliminary screening was first performed using Lipinski’s rule of 5, a criterion for selecting small, druggable molecules. DS LibDock, a rapid, rigid docking extension of the BIOVIA Discovery Studio Client 2020 software, was subsequently used to perform quick and efficient docking by pinpointing hotspots near the protein-binding site domain. These sites were then used to direct the drugs, thereby achieving rigid alignment of the ligand conformations and generating favorable interactions. A final energy minimization was performed to allow flexibility in ligand poses, and the highest-scoring poses were saved.

#### 2.5.2. Flexible Docking Analysis

A second layer of screening used a more time-consuming and computationally intensive extension of BIOVIA’s Discovery Studio Client 2020, known as CDOCKER. CDOCKER is a docking parameter that implements a DS CHARMm-based grid docking method. Ligand conformations are generated via high-temperature molecular dynamics (MD) followed by refinement, in which the protein’s binding residues are made flexible through simulated annealing in MD. CDOCKER enables the rapid calculation of a physics-based scoring function by using the DS CHARMm energy of the docked complex, serving as a flexible docking tool for both small molecules and macromolecules. The DS CHARMm force field is ideal for high-throughput analysis of large ligand sets, producing docked conformations with exceptional precision.

#### 2.5.3. ADMET Analysis

BIOVIA Discovery Studio Client 2020 was used to perform Absorption, Distribution, Metabolism, Excretion, and Toxicity (ADMET) analysis and to measure the exact pharmacokinetic properties of protein-drug interactions. The ADMET descriptors protocol was employed, using the QSAR model’s estimated range of training sets to predict ADMET properties for test sets or small molecules. Blood–brain barrier (BBB) penetration, cytochrome P450 (CYP450) 2D6 inhibition, hepatotoxicity, human intestinal absorption (HIA), plasma protein binding, and other parameters were all computed in this analysis, followed by an Ames test to determine potential mutagenicity or genotoxicity.

### 2.6. Cell Culture

Human liver cancer cell lines, SK Hep-1, were obtained from ATCC and cultured in Modified Eagle’s Medium (EMEM, ATCC), supplemented with 10% fetal bovine serum (FBS) and incubated at 37 °C under 5% CO_2_.

*Drug procurement:* Sorafenib, malondialdehyde HCl, mercaptoethanol, glycine, parachlorophenol, methoxyamine, and ethanolamine were purchased from Sigma-Aldrich (St. Louis, MO, USA).

#### 2.6.1. RNA Isolation

Total cell RNA was extracted from 100 mm plates containing 1 × 10^6^ log-phase cells using TRIzol (Invitrogen, Carlsbad, CA, USA) and isolated according to the manufacturer’s protocol. Homogenates were then transferred to RNase-free tubes, and RNA was isolated according to the manufacturer’s protocol. RNA quality was assessed using Nanodrop agarose gel electrophoresis to visualize the 5S, 18S, and 28S bands.

#### 2.6.2. qRT-PCR

Total RNA was isolated as described above, and equal amounts of RNA were reverse-transcribed into complementary DNA (cDNA) using the Thermo Fisher kit according to the manufacturer’s instructions (Thermo Fisher, Vilnius, LT, USA). The resulting cDNA was used for quantitative real-time PCR with SYBR Green PCR Master Mix (Biorad) and YBX1-specific primers. Reactions were run in technical quadruplicates, with appropriate controls. Relative gene expression was calculated using the 2^−ΔΔCt^ method, with Ct values normalized to an internal housekeeping gene (ACTB) and expressed relative to the control group (Supplementary Table S2).

#### 2.6.3. Western Blot

Cells were harvested and lysed in RIPA buffer supplemented with protease and phosphatase inhibitors. Protein levels were quantified using the Bradford protein estimation kit with bovine serum albumin (BSA) as the standard, according to the manufacturer’s protocol. Equal aliquots of protein (10 µg/lane) were then electrophoresed through a PAGE gel under reducing conditions (4–12% sodium dodecyl sulfate–PAGE Bis-Tris gels’ MOPS buffer system, Invitrogen (NuPAGE-MOPS)). Gels were blotted onto nitrocellulose membranes, blocked with 5% milk, and incubated with the primary antibody YBX1 (Supplementary Table S1) overnight. Afterward, they were washed with TBST, and membranes were incubated with fluorescence-labeled secondary antibodies for 1 h. Blots were washed with TBST and developed using the Odyssey CLX LI-COR detection system.

## 3. Results

### 3.1. Functional Validation of Conserved Binding Domain

**Figure 1:**
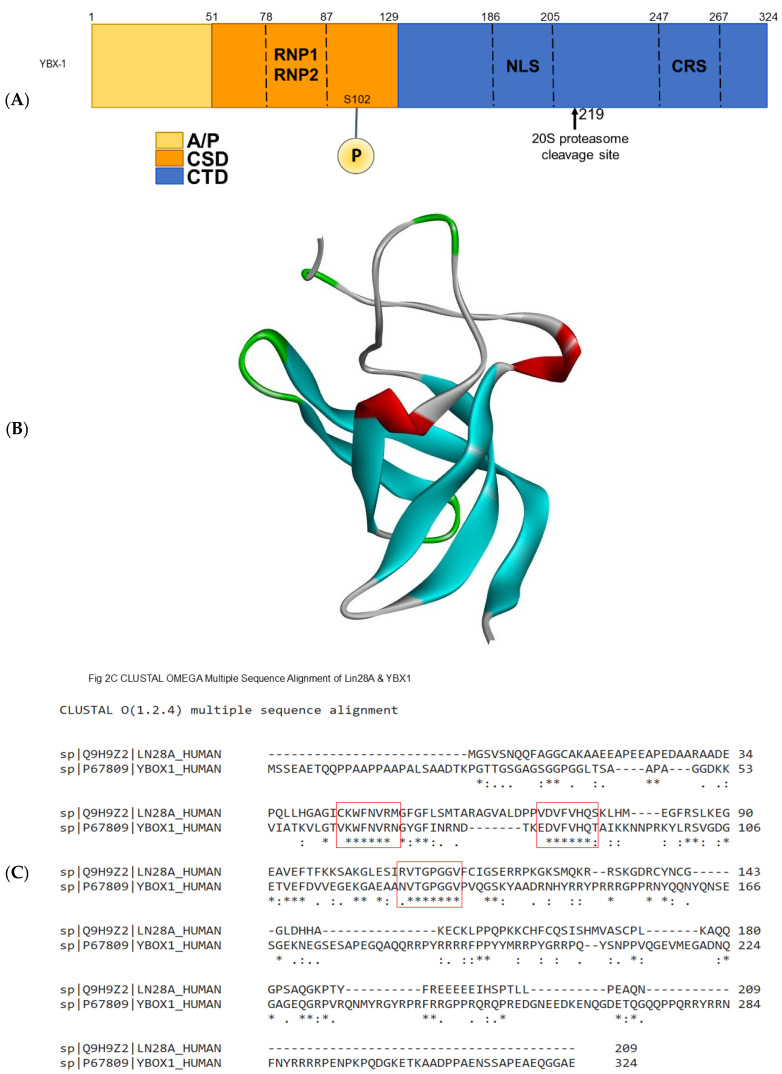
Schematic representation of the YBX1 protein: (**A**) This diagram highlights the alanine/proline (A/P)-rich domain (residues 1–50), the evolutionarily conserved cold shock domain (CSD) (residues 51–129), and the C-terminal domain (CTD) (residues 130–324), which features four positively charged arginine-rich clusters alternating with four negatively charged amino acid cluster residues. The RNA-binding RNP-1 and RNP-2-like motifs span residues 70–77 and 84–87, respectively. Phosphorylation of S102 induces nuclear translocation of the protein. The nuclear localization signal (NLS) spans residues 186–205. The cytoplasmic retention site (CRS) spans residues 247–267. (**B**) 3D crystal structure of YBX1 CSD. This shows the 3D crystal structure of the YBX1 CSD, taken from RSCB PDB (PDB ID: 6LMR). The YBX1 CSD crystal structure is shown in a ribbon representation with rainbow coloring from blue (N-terminus) to red (C-terminus), indicating the amino acid sequence. Blue → N-terminal region (beginning of the protein sequence). Cyan/Green → Middle portion of the sequence. Yellow/Orange → Later regions. Red → C-terminal region (end of the protein sequence). (**C**). CLUSTAL OMEGA Multiple-Sequence Alignment of Lin28A & YBX1. The multiple-sequence alignment between Lin28A and YBX1 shows three conserved homologous domains shared between the two proteins, as indicated by the red boxes. * = fully conserved regions. : = strong, similar properties. . = weak, similar properties.

A literature search was conducted to identify transcription factors other than YBX1 with a homologous binding domain, thereby providing functional validation of YBX1’s conserved nucleic acid-binding domain (cold shock domain). The schematic of the YBX1 domain is shown in Figure 1A. The cold shock domain structure is shown in Figure 1B. Lin28 is a transcription factor that also contains this highly conserved domain. The CLUSTAL Omega Multiple-Sequence Alignment tool was used to analyze and compare the consensus sequences between the two proteins and three conserved regions identified within their respective cold shock domains (Figure 1C).

### 3.2. Preparation of Macromolecules

**Figure 2:**
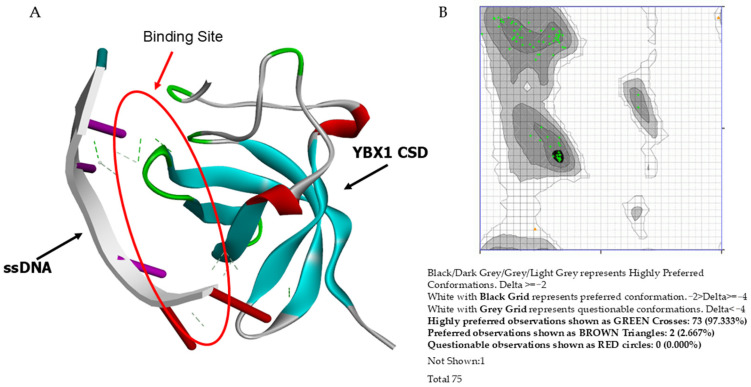
YBX1 binding domain model and verification: (**A**) Image generated by Discovery Studio depicting the interaction of the YBX1 binding domain (PDB ID: 6LMR) with single-stranded DNA (ssDNA). (**B**) The Ramachandran plot used to assess the torsional angle viability of YBX1 (PDB: 6LMR) via the Ramachandran Plot Server.

The YBX1 protein structure was obtained from the RCSB Protein Data Bank (Figure 2A). PDB ID 6LMR shows that the YBX1 protein’s cold shock domain (binding domain) binds single-stranded DNA (ssDNA) as a transcription factor. The protein model was verified by downloading the PDB file and running it through the Z-Lab Ramachandran Plot Server. A Ramachandran plot with 97.33% in the highly preferred regions (over 90% as expected) was observed (Figure 2B). The structure was selected and edited in Discovery Studio to remove HETATM entries. An energy minimization step was performed to remove any steric collisions, using the following parameters: 1000 steepest descent steps with a descent size of 0.02 Å and 1000 conjugate gradient steps with a step size of 0.02 Å for conjugate gradient minimization.

### 3.3. Multi-Layered High-Throughput Virtual Screening

**Figure 3:**
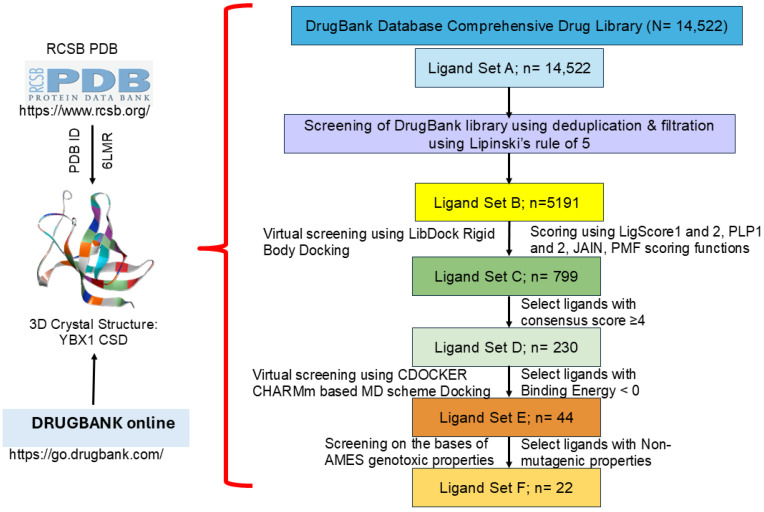
High-throughput virtual screening (HTVS). Schematic showing the systematic multi-layered high-throughput virtual screening (HTVS) of the DrugBank compounds against YBX1 (PDB ID: 6LMR). https://www.rcsb.org/, https://go.drugbank.com/.

The DrugBank library of experimental and approved drugs was used as the primary dataset. In the HTVS process, several screening layers were performed to identify the best drug that binds to and inhibits the YBX1 protein (Figure 3). In the first layer, Lipinski’s rule of 5 was applied, reducing Ligand Set A (14,522 molecules) to Ligand Set B (5191 molecules). Ligand Set B exhibits druggable properties and was advanced to the next screening layer using rigid, fast docking protocols via the DS LibDock tool (v20.1). This further reduced Ligand Set B from 5191 molecules to Ligand Set C (799 molecules). Additional parameters were applied to the screening in the form of consensus scores (LigScore 1 and 2, PLP1 and 2, JAIN, and PMF) [26]. Of the six consensus scores, four were used as the screening threshold. This eliminated 569 molecules, leaving only 230 molecules in Ligand Set D that qualified for the next layer of screening with DS CDOCKER. For this, a CHARMM-based force-field docking analysis of the 230 molecules from Ligand Set D was performed to narrow it down to 44 molecules as the best fit for Ligand Set E, using negative binding energies as the selection criterion for this phase. After CHARMM-based docking, 44 drugs remained and were further screened using AMES genotoxicity parameters, excluding those that showed mutagenic properties in the AMES test, to obtain the final Ligand Set F. From the final 22 compounds, an extensive literature search was conducted to determine whether any had been tested in any cancer types or as YBX1 inhibitors (Table 1, Figures S1–S22). Based on these parameters, malonaldehyde, mercaptoethanol, glycine, parachlorophenol, methoxyamine, and ethanolamine showed the most significant potential for further investigation.

### 3.4. ADMET Analysis

**Figure 4:**
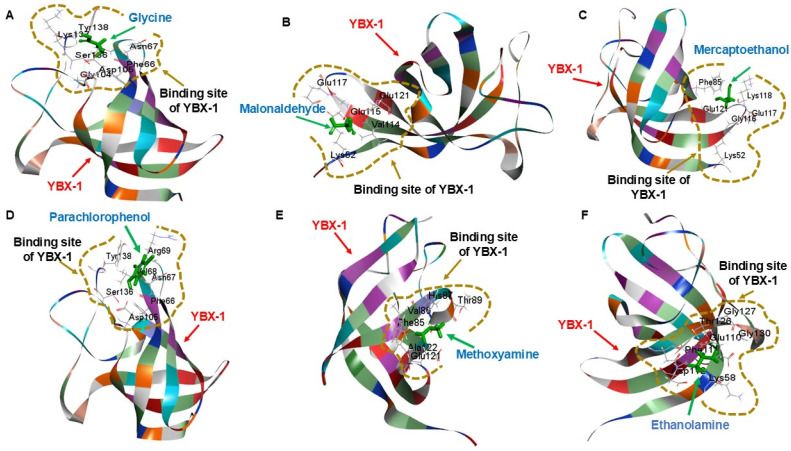
Binding pattern analysis for the top 6 potentially inhibiting compounds of YBX1. Binding pattern analysis images (generated by Discovery Studio Client) between (**A**) glycine (DrugBank ID: DB00145; PubChem ID: 750), (**B**) malonaldehyde (DrugBank ID: DB03057; PubChem ID: 10964), (**C**) mercaptoethanol (DrugBank ID: DB03345; PubChem ID: 1567), (**D**) parachlorophenol (DrugBank ID: DB13154; PubChem ID: 4684), (**E**) methoxyamine (DrugBank ID: DB06328; PubChem ID: 4113), and (**F**) ethanolamine (DrugBank ID: DB03994; PubChem ID: 700) and the YBX 1 CSD (PDB ID: 6LMR).

The ADMET descriptor protocol used to estimate a range of ADMET-related properties for the final screening included computed parameters for aqueous solubility at 25 °C, blood–brain barrier penetration (BBB), cytochrome P450 (CYP450) 2D6 inhibition, hepatotoxicity, and plasma protein binding (chances of drugs being bound to carrier proteins within the blood). The completed ADMET analysis indicated that none of the 22 final compounds from the HTVS were likely to bind plasma proteins or be metabolized by CYP2D6. All but two compounds—glycine and glycolic acid—showed some degree of hepatotoxicity. Glycine and glycolic acid had predicted solubilities of 5.52 × 10^2^ g/L and 6.08 × 10^2^ g/L, respectively, and neither was likely to cross the blood–brain barrier (Table 2). Among the 22 compounds, malonaldehyde, mercaptoethanol, glycine, parachlorophenol, methoxyamine, and ethanolamine, used for bonding pattern analysis, were found to demonstrate the greatest potential for further investigation (Figure 4).

### 3.5. Glycine in Drug Resistance

**Figure 5:**
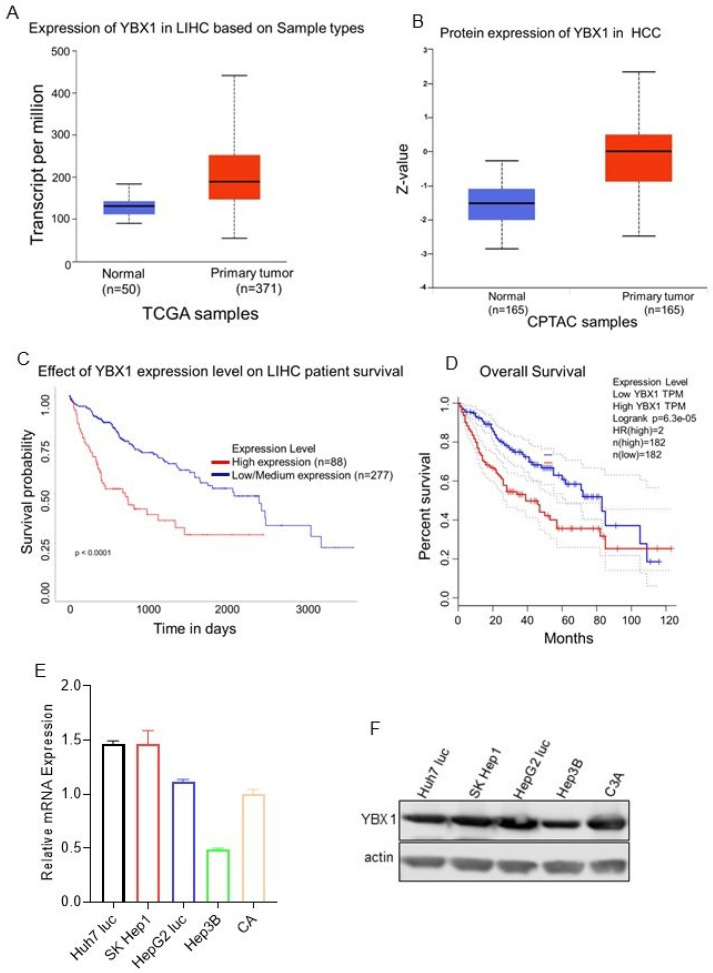

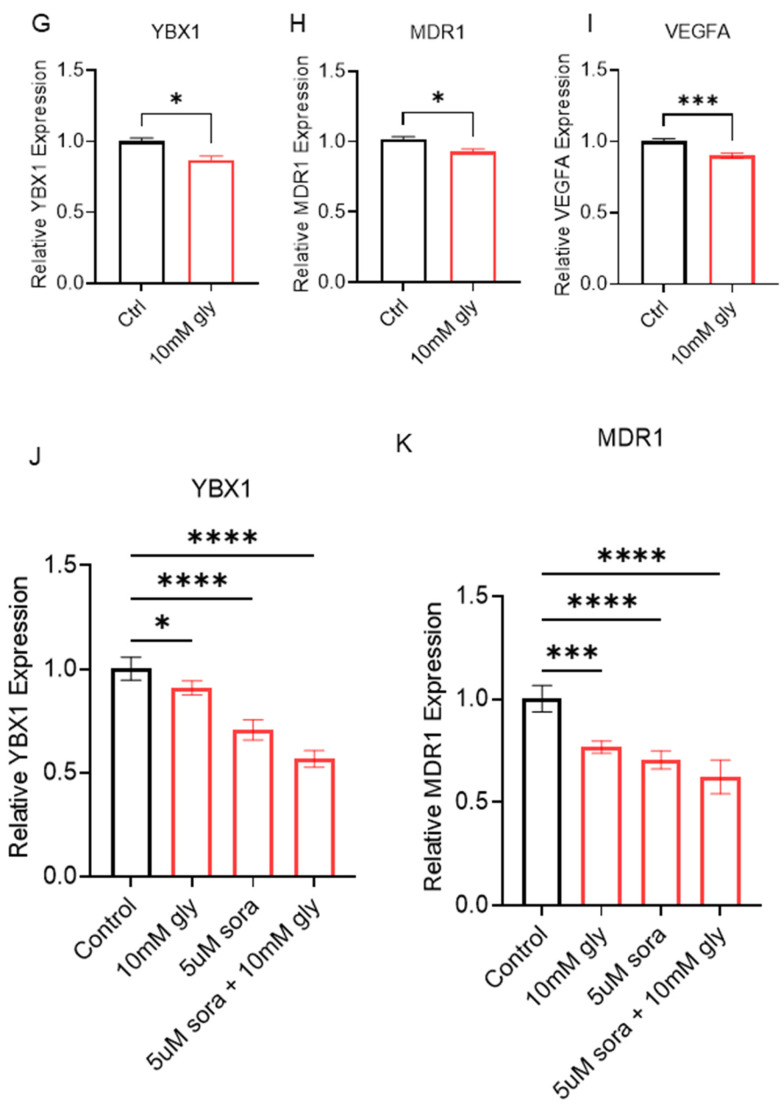
TCGA Analysis of YBX1 Expression in HCC: (**A,B**) YBX1 expression is notably higher in liver cancer (LIHC) samples compared to normal liver tissue, and increased protein levels are likewise seen in tumor samples relative to normal tissue. (**C**,**D**) Survival and overall survival are also lower in patients with high YBX1 expression (TCGA data). (**E**,**F**) Expression of YBX1 in hepatocellular carcinoma cells: RT-qPCR and Western blot. (**G**–**I**) SK Hep1 cells were treated with 10 mM glycine, and the YBX1 and downstream targets were observed. (**J**) SK Hep1 cells were treated with glycine, sorafenib, and glycine with sorafenib, and RNA levels of YBX were observed. (**K**) SK Hep1 cells were treated with glycine, sorafenib, and glycine with sorafenib, and RNA levels of MDR1 were measured. Data are presented as mean ± SD. n = 3 per condition. One-way ANOVA followed by Tukey’s multiple-comparison test. (Data are shown as mean ± SEM. The comparison between the two groups was performed using an unpaired two-sided *t*-test (* *p*-value < 0.05, *** *p*-value < 0.001, **** *p*-value < 0.0001).

TCGA analysis revealed that YBX1 is highly expressed at both RNA and protein levels in various cancers, including HCC (Figure 5A,B). Clinical patient data indicate that survival and overall survival rates are significantly lower in patients with YBX1 overexpression (Figure 5C,D). Taken together, we conducted a comparative study of YBX1 mRNA expression in hepatocarcinoma cell line models, showing YBX1 upregulation (Figure 5E). This trend was corroborated at the protein level, with elevated YBX1 expression observed in five hepatocarcinoma cell lines (Figure 5F). These results indicate that YBX1 is implicated in hepatocellular carcinoma progression and liver metastasis. SK Hep1 cells were treated with 10 mM glycine alone for 24 h. Interestingly, YBX1 is moderately downregulated, and genes associated with drug resistance, including MDR1 and VEGF, are also downregulated. We analyzed glycine treatment and found downregulation of YBX1 and associated drug resistance genes, MDR1 and VEGF (Figure 5G–I). Furthermore, SK Hep1 cells were treated with glycine, sorafenib, and a combination of glycine and sorafenib, and the RNA levels of YBX1 were assessed. A combination of sorafenib and glycine treatment resulted in a significant decrease in YBX1 and MDR1 levels (Figure 5J,K). These preliminary results suggest that a low concentration of glycine, when combined with sorafenib, helps reduce sorafenib resistance by lowering the expression of YBX1, MDR1, and VEGF in hepatocellular carcinoma.

## 4. Discussion

Cancer is the leading cause of death worldwide and a significant public health issue. Targeting RNA–protein interactions offers a promising approach to cancer prevention. YBX1 is highly expressed in various malignant tumors, including HCC, making it a potential therapeutic target. Here, we identified the number of YBX1 CSD-targeted therapeutic molecules that inhibit YBX1, suggesting YBX1 CSD as a potential target for new drug development. In this study, we determined the crystal structures of YBX1 CSD-binding sites and inhibitors using several in silico methods. YBX1 is upregulated in many cancers, including HCC. Its structure is not available in the Protein Data Bank (PDB) because YBX1 contains a CSD. However, comparative structural studies of other cold shock domain family members have shown that the YBX1 CSD in mammalian Lin28a proteins and the human Lin28a CSD (PDB code 5UDZ) are structurally similar [27]. We further screened the DrugBank library for the number of binding partners or inhibitors of YBX1.

In this work, we also identified 22 potential YBX1 targets and inhibitors using a multi-layered, high-throughput virtual screening approach. Following this rigorous screening, an extensive literature search helped finalize six compounds for further investigation, including malonaldehyde, mercaptoethanol, glycine, parachlorophenol, methoxyamine, and ethanolamine. Among these, many have been verified in preclinical models or clinical trials across various cancers. In particular, the amino acid glycine, which shows potential as an inhibitor of YBX1’s binding domain, is especially exciting.

Malonaldehyde is a highly reactive and toxic aldehyde formed by lipid peroxidation of polyunsaturated fatty acids via reactive oxygen species and by the breakdown of prostaglandins via cyclooxygenase. Malonaldehyde has been shown by Eerejuwa et al. to potentially inhibit cancer growth or induce cancer cell death [28]. It was found that it enhanced the cytotoxicity of doxorubicin, vincristine, and fludarabine against leukemic cells; increased the sensitivity of colorectal adenocarcinoma cells to radiotherapy; inhibited tumor growth in breast and mammary carcinoma cells; and induced DNA fragmentation and apoptosis in colon cancer cells [28]. Mercaptoethanol is a water-soluble, sulfur-based antioxidant with an unpleasant odor that reduces and cleaves disulfide bonds, prevents protein oxidation, and degrades ribonucleases. Click found that in mouse models treated with 2-mercaptoethanol, lifetime prevention of the development of spontaneous and radiation-induced mammary cancers was possible and even suggested a slowed or delayed progression in mouse models with liver tumors [29].

Parachlorophenol is a water-soluble, corrosive, and slightly toxic phenol that uncouples oxidative phosphorylation. Tuffin et al. found that HepG2 liver cancer cells treated with 4-monochlorophenol in culture showed a significant increase in oxidative stress and a decrease in ATP production [30]. Methoxyamine is a water-soluble, volatile compound used as an oral small-molecule inhibitor of base excision repair and as an inhibitor of topoisomerase II to reverse resistance to chemotherapy and improve radiation therapy. The results of a Phase I clinical trial of TRC102 (methoxyamine HCl), conducted by Coyne et al., in combination with temozolomide (an alkylating agent), showed that 4 out of 51 patients with various cancer types had partial responses, while another 13 out of 51 experienced stable disease, both with manageable side-effect profiles [31]. Another Phase I clinical trial by Caimi et al. combined methoxyamine with fludarabine in patients with advanced hematologic malignancies and found that this combination was associated with increased DNA damage. In contrast, hematologic toxicity was comparable to that of fludarabine alone [23]. Furthermore, Pezuk et al. demonstrated that exposure to methoxyamine in pediatric medulloblastoma cell lines treated with ionizing radiation and chemotherapy significantly reduced cell proliferation and increased apoptosis [32]. Ethanolamine serves as a precursor to phosphatidylethanolamine (lecithin) and functions as a surfactant, fluorometric reagent, and agent for removing CO_2_ and H2S from various gases. Saxena et al. demonstrated the potential of a nontoxic oral formulation of monoethanolamine for prostate cancer treatment in preclinical models, showing reduced cell proliferation in cell cultures and in mouse models [33].

Glycine is the smallest and simplest amino acid, essential for forming alpha-helices in secondary protein structures and functioning as an inhibitory neurotransmitter in the spinal cord. This makes it the most promising among the six compounds. In a review of the physicochemical properties of anticancer peptides, Chiangjian et al. reported that glycine may play a role in membrane interactions and the conformational flexibility of cancer cells [34]. Additionally, Maneikyte et al. demonstrated that glycine significantly reduced tumor volume and decreased tumor microvascular density in in vivo models of colorectal liver metastases, without interfering with the anti-tumor effects of chemotherapy [35,36]. Previous studies have also demonstrated that glycine plays a crucial role in regulating angiogenesis by reducing VEGFA levels at both the mRNA and protein levels in HCC. Rose Marden et al. showed that glycine inhibits HCC induction in toxic rat models. Furthermore, glycine can reduce chemotherapy-induced liver damage through mechanisms dependent on Kupffer cells in non-malignant liver tissue [37]. Serine–glycine biosynthesis regulatory genes (PHGDH, PSAT1, PSPH, SHMT1, and SHMT2) also play a vital role in cancer cell proliferation [38].

## 5. Conclusions

In this study, we used HTVS in BIOVIA Discovery Studio Client 2020 to identify YBX1 inhibitors and refine the potential inhibitors through computational and in vitro studies. We also demonstrated an alternative approach to this method, applicable to proteins whose 3D crystal structures are unavailable in the RCSB PDB. Impairing YBX1 expression could sensitize sorafenib-resistant cells [8,39]. Our results suggest that low concentrations of glycine, combined with sorafenib, may help reduce sorafenib resistance in hepatocellular cancers. Our data indicate that treatment with 10 mM glycine moderately decreases YBX1 expression in HCC cells, accompanied by reductions in downstream targets, including MDR1 and VEGFA. Furthermore, a combination of sorafenib and glycine treatment resulted in a significant decrease in YBX1 and MDR1 levels compared with sorafenib alone. This confirms the role of glycine in angiogenesis, as reported by Bruns et al., and suggests an unknown role in cellular growth, proliferation, and apoptosis, as well as in cellular adhesion, migration, invasion, and metastasis in colorectal cancer [18]. Additional studies are needed to confirm the role of glycine in sorafenib resistance in HCC and to further elucidate its mechanism.

## Figures and Tables

**Table 1:** HTVS finalized compound list.

S.No.	Protein Name	DrugBank ID	PubChem ID	Mol Wt.	Mol.Formula	Status of Drug	Generic Name	Binding Energy	CDocker Energy	LibDock Score	Protein Confirmation
1	YBX1 (pdb id: 6lmr)	DB14511	175	59.0133	C_2_H_3_O_2_	experimental	Acetate	−148.296	−5.7735	25.3983	74
2	YBX1 (pdb id: 6lmr)	DB03057	10964	72.0211	C_3_H_4_O_2_	experimental	Malonaldehyde	−74.3094	−11.5669	21.1194	54
3	YBX1 (pdb id: 6lmr)	DB02825	3396559	78.9949	CH_4_O_2_P	experimental	Methylphosphinate	−68.3031	−19.678	15.1432	75
4	YBX1 (pdb id: 6lmr)	DB03729	162636	149.059	C_7_H_7_N_3_O	experimental	2-Amino-1H-benzimidazol-5-ol	−55.8001	9.16909	24.9335	65
5	YBX1 (pdb id: 6lmr)	DB13343	72139	167.988	C_7_H_4_O_3_S	experimental	Tioxolone	−55.2934	−0.864032	27.0726	94
6	YBX1 (pdb id: 6lmr)	DB03345	1567	78.0139	C_2_H_6_OS	experimental	Mercaptoethanol	−45.9513	−15.4157	19.9068	76
7	YBX1 (pdb id: 6lmr)	DB02297	118458	129.009	C_4_H_4_ClN_3_	experimental	2-Amino-6-Chloropyrazine	−44.5632	2.18912	45.221	83
8	YBX1 (pdb id: 6lmr)	DB01957	7933	128.003	C_6_H_5_ClO	experimental	3-Chlorophenol	−39.6083	4.16052	41.1874	47
9	YBX1 (pdb id: 6lmr)	DB04261	57418154	61.0164	CH_3_NO_2_	experimental	Carbamic Acid	−33.4564	10.4189	14.9349	37
10	YBX1 (pdb id: 6lmr)	DB02806	8019	76.0524	C_3_H_8_O_2_	experimental	2-Methoxyethanol	−33.2482	−25.0526	38.3834	83
11	YBX1 (pdb id: 6lmr)	DB03175	1031	60.0575	C_3_H_8_O	approved	Propyl alcohol	−33.1615	−23.6707	25.6637	36
12	YBX1 (pdb id: 6lmr)	DB00898	702	46.0419	C_2_H_6_O	approved	Ethanol	−31.8429	−6.7718	25.8371	63
13	YBX1 (pdb id: 6lmr)	DB00145	750	75.032	C_2_H_5_NO_2_	approved; nutraceutical; vet_approved	**Glycine**	−31.7796	−13.9803	31.4454	86
14	YBX1 (pdb id: 6lmr)	DB14189	3301	60.0687	C_2_H_8_N_2_	approved; experimental	Ethylenediamine	−31.7246	−13.7926	32.9177	91
15	YBX1 (pdb id: 6lmr)	DB13154	4684	128.003	C_6_H_5_ClO	approved	Parachlorophenol	−30.3799	8.2681	28.6625	76
16	YBX1 (pdb id: 6lmr)	DB06328	4113	47.0371	CH_5_NO	investigational	Methoxyamine	−29.2921	6.30856	23.2366	46
17	YBX1 (pdb id: 6lmr)	DB03994	700	61.0528	C_2_H_7_NO	experimental	Ethanolamine	−28.2542	−12.1253	33.4183	12
18	YBX1 (pdb id: 6lmr)	DB02646	79124	59.0371	C_2_H_5_NO	experimental	Nitrosoethane	−19.7283	−8.94346	27.4351	83
19	YBX1 (pdb id: 6lmr)	DB12529	946	45.9929	NO_2_	approved; investigational	Nitrite	−19.2628	6.63338	7.4109	53
20	YBX1 (pdb id: 6lmr)	DB04053	183145	62.9636	O_2_P	experimental	Hypophosphite	−16.6614	9.50035	7.242	9
21	YBX1 (pdb id: 6lmr)	DB03085	3698251	76.016	C_2_H_4_O_3_	approved; investigational	Glycolic acid	−14.7563	−25.8587	34.3981	4
22	YBX1 (pdb id: 6lmr)	DB01968	17754199	75.0143	C_2_H_5_NS	experimental	2-Thioethenamine	−14.1409	−12.9928	19.6568	36

List of compounds finalized from the DrugBank library screening of 14,522 compounds. Compounds are arranged in order to increase binding energy (kcal/mol).

**Table 2:** ADMET descriptors of HTVS compound list.

S.No.	Generic Name	ALogPS Solubility	BBB Level	CYP2D6 Prediction	Hepatotoxicity Prediction	Plasma Protein Binding
1	Acetate	4.90 × 10^2^ g/L	4	FALSE	TRUE	FALSE
2	Malonaldehyde	2.41 × 10^2^ g/L	3	FALSE	TRUE	FALSE
3	Methylphosphinate	3.94 × 10^2^ g/L	4	FALSE	TRUE	FALSE
4	2-Amino-1H-benzimidazol-5-ol	—	3	FALSE	TRUE	FALSE
5	Tioxolone	3.71 × 10^0^ g/L	2	FALSE	TRUE	FALSE
6	Mercaptoethanol	2.92 × 10^1^ g/L	4	FALSE	TRUE	FALSE
7	2-Amino-6-Chloropyrazine	5.59 × 10^1^ g/L	3	FALSE	TRUE	FALSE
8	3-Chlorophenol	1.46 × 10^1^ g/L	1	FALSE	TRUE	TRUE
9	Carbamic Acid	3.79 × 10^2^ g/L	4	FALSE	TRUE	FALSE
10	2-Methoxyethanol	8.12 × 10^2^ g/L	4	FALSE	TRUE	FALSE
11	Propyl alcohol	3.91 × 10^2^ g/L	2	FALSE	TRUE	FALSE
12	Ethanol	5.79 × 10^2^ g/L	4	FALSE	TRUE	FALSE
13	Glycine	5.52 × 10^2^ g/L	4	FALSE	FALSE	FALSE
14	Ethylenediamine	5.60 × 10^2^ g/L	4	FALSE	TRUE	FALSE
15	Parachlorophenol	1.40 × 10^1^ g/L	1	FALSE	TRUE	TRUE
16	Methoxyamine	6.00 × 10^2^ g/L	4	FALSE	TRUE	FALSE
17	Ethanolamine	8.49 × 10^2^ g/L	4	FALSE	TRUE	FALSE
18	Nitrosoethane	4.33 × 10^1^ g/L	2	FALSE	TRUE	FALSE
19	Nitrite	—	3	FALSE	TRUE	FALSE
20	Hypophosphite	—	4	FALSE	TRUE	FALSE
21	Glycolic acid	6.08 × 10^2^ g/L	4	FALSE	FALSE	FALSE
22	2-Thioethenamine	1.77 × 10^1^ g/L	4	FALSE	TRUE	FALSE

TRUE = positive/yes; FALSE = negative/no.

## Data Availability

The original contributions presented in this study are included in the article/Supplementary Materials. Further inquiries can be directed to the corresponding author.

## Supplementary Materials

The following supporting information can be downloaded at: https://www.mdpi.com/article/10.3390/biomedicines14030545/s1, Figure S1: HTVS compound Acetate binding pattern analysis with YBX1. Figure S2: Malonaldehyde binding pattern analysis with YBX1. Figure S3: Methylphosphinate binding pattern analysis with YBX1. Figure S4: 2-Amino-1H-benzimidazole-5-ol binding pattern analysis with YBX1. Figure S5: Tioxolone binding pattern analysis with YBX1. Figure S6: Mercaptoethanol binding pattern analysis with YBX1. Figure S7: 2-Amino-6-Chloropyrazine binding pattern analysis with YBX1. Figure S8: 3-Chlorophenol binding pattern analysis with YBX1. Figure S9: Carbamic Acid binding pattern analysis with YBX1. Figure S10: 2-Methoxyethanol binding pattern analysis with YBX1. Figure S11: Propyl Alcohol binding pattern analysis with YBX1. Figure S12: Ethanol binding pattern analysis with YBX1. Figure S13: Glycine binding pattern analysis with YBX1. Figure S14: Ethylenediamine binding pattern analysis with YBX1. Figure S15: Parachlorophenol binding pattern analysis with YBX1. Figure S16: Methoxyamine binding pattern analysis with YBX1. Figure S17: Ethanolamine binding pattern analysis with YBX1. Figure S18: Nitrosoethane binding pattern analysis with YBX1. Figure S19: Nitrite binding pattern analysis with YBX1. Figure S20: Hypophosphite binding pattern analysis with YBX1. Figure S21: Glycolic Acid binding pattern analysis with YBX1. Figure S22: 2-Thioethenamine binding pattern analysis with YBX1. Table S1: Details of antibodies used in the study. Table S2: RT-PCR Primers used in this study.

## Abbreviations

ABC	ATP-binding cassette
ADMET	Absorption, distribution, metabolism, excretion, and toxicity
BBB	Blood–brain barrier penetration
CSD	Cold shock domain
EMT	Epithelial–mesenchymal transition
HTVS	High-throughput virtual screening
MD	Molecular dynamics
MDR1	Multi-drug resistant 1
PDB	Protein Data Bank
PHGDH	Phosphoglycerate dehydrogenase
PSAT1	Phosphoserine aminotransferase 1
PSPH	Phosphoserine phosphatase
TKIs	Tyrosine-kinase inhibitors
VEGFA	Vascular endothelial growth factor A
YBX1	Y box binding protein

## References

[1] Siegel R.L., Kratzer T.B., Giaquinto A.N., Sung H., Jemal A. (2025). Cancer statistics, 2025. CA Cancer J. Clin..

[2] Mittal S., El-Serag H.B. (2013). Epidemiology of hepatocellular carcinoma: Consider the population. J. Clin. Gastroenterol..

[3] Gomaa A.I., Khan S.A., Toledano M.B., Waked I., Taylor-Robinson S.D. (2008). Hepatocellular carcinoma: Epidemiology, risk factors and pathogenesis. World J. Gastroenterol..

[4] Marin J.J.G., Macias R.I.R., Monte M.J., Romero M.R., Asensio M., Sanchez-Martin A., Cives-Losada C., Temprano A.G., Espinosa-Escudero R., Reviejo M. (2020). Molecular Bases of Drug Resistance in Hepatocellular Carcinoma. Cancers.

[5] Haider T., Pandey V., Banjare N., Gupta P.N., Soni V. (2020). Drug resistance in cancer: Mechanisms and tackling strategies. Pharmacol. Rep..

[6] Leonard G.D., Fojo T., Bates S.E. (2003). The role of ABC transporters in clinical practice. Oncologist.

[7] Kuwano M., Shibata T., Watari K., Ono M. (2019). Oncogenic Y-box binding protein-1 as an effective therapeutic target in drug-resistant cancer. Cancer Sci..

[8] Kwabiah D., Nagati V., Tripathi M.K. (2025). Transcription factor YBX1 orchestrates drug resistance and tumor progression in HCC. Drug Discov. Today.

[9] Li J., Chen H., Bai L., Tang H. (2025). RNF115 upregulation by YBX1-dependent m5C modification promotes hepatocellular carcinoma progression. npj Precis. Oncol..

[10] Gopal S.K., Greening D.W., Mathias R.A., Ji H., Rai A., Chen M., Zhu H.J., Simpson R.J. (2015). YBX1/YB-1 induces partial EMT and tumourigenicity through secretion of angiogenic factors into the extracellular microenvironment. Oncotarget.

[11] Alkrekshi A., Wang W., Rana P.S., Markovic V., Sossey-Alaoui K. (2021). A comprehensive review of the functions of YB-1 in cancer stemness, metastasis and drug resistance. Cell. Signal..

[12] Chandrashekar D.S., Karthikeyan S.K., Korla P.K., Patel H., Shovon A.R., Athar M., Netto G.J., Qin Z.S., Kumar S., Manne U. (2022). UALCAN: An update to the integrated cancer data analysis platform. Neoplasia.

[13] Chandrashekar D.S., Bashel B., Balasubramanya S.A.H., Creighton C.J., Ponce-Rodriguez I., Chakravarthi B., Varambally S. (2017). UALCAN: A Portal for Facilitating Tumor Subgroup Gene Expression and Survival Analyses. Neoplasia.

[14] Bao M.H.-R., Wong C.C.-L. (2021). Hypoxia, Metabolic Reprogramming, and Drug Resistance in Liver Cancer. Cells.

[15] Mooli R.G.R., Mukhi D., Watt M., Nagati V., Reed S.M., Gandhi N.K., Oertel M., Ramakrishnan S.K. (2024). Hypoxia-Inducible Factor-2α Promotes Liver Fibrosis by Inducing Hepatocellular Death. Int. J. Mol. Sci..

[16] Chao H.M., Huang H.X., Chang P.H., Tseng K.C., Miyajima A., Chern E. (2017). Y-box binding protein-1 promotes hepatocellular carcinoma-initiating cell progression and tumorigenesis via Wnt/β-catenin pathway. Oncotarget.

[17] Khan M.I., Adhami V.M., Lall R.K., Sechi M., Joshi D.C., Haidar O.M., Syed D.N., Siddiqui I.A., Chiu S.-Y., Mukhtar H. (2014). YB-1 expression promotes epithelial-to-mesenchymal transition in prostate cancer that is inhibited by a small molecule fisetin. Oncotarget.

[18] Bruns H., Kazanavicius D., Schultze D., Saeedi M.A., Yamanaka K., Strupas K., Schemmer P. (2016). Glycine inhibits angiogenesis in colorectal cancer: Role of endothelial cells. Amino Acids.

[19] Rose M.L., Cattley R.C., Dunn C., Wong V., Li X., Thurman R.G. (1999). Dietary glycine prevents the development of liver tumors caused by the peroxisome proliferator WY-14,643. Carcinogenesis.

[20] Bruns H., Petrulionis M., Schultze D., Al Saeedi M., Lin S., Yamanaka K., Ambrazevičius M., Strupas K., Schemmer P. (2014). Glycine inhibits angiogenic signaling in human hepatocellular carcinoma cells. Amino Acids.

[21] Laskowski R.A., MacArthur M.W., Moss D.S., Thornton J.M. (1993). PROCHECK: A program to check the stereochemical quality of protein structures. J. Appl. Crystallogr..

[22] Madeira F., Park Y.M., Lee J., Buso N., Gur T., Madhusoodanan N., Basutkar P., Tivey A.R.N., Potter S.C., Finn R.D. (2019). The EMBL-EBI search and sequence analysis tools APIs in 2019. Nucleic Acids Res..

[23] Knox C., Wilson M., Klinger C.M., Franklin M., Oler E., Wilson A., Pon A., Cox J., Chin N.E.L., Strawbridge S.A. (2024). DrugBank 6.0: The DrugBank Knowledgebase for 2024. Nucleic Acids Res..

[24] Wishart D.S., Feunang Y.D., Guo A.C., Lo E.J., Marcu A., Grant J.R., Sajed T., Johnson D., Li C., Sayeeda Z. (2018). DrugBank 5.0: A major update to the DrugBank database for 2018. Nucleic Acids Res..

[25] Dhasmana A., Raza S., Jahan R., Lohani M., Arif J.M., Khan A.M.S., Ahmad I., Chattopadhyay D. (2019). Chapter 19—High-Throughput Virtual Screening (HTVS) of Natural Compounds and Exploration of Their Biomolecular Mechanisms: An In Silico Approach. New Look to Phytomedicine.

[26] Dhasmana A., Kashyap V.K., Dhasmana S., Kotnala S., Haque S., Ashraf G.M., Jaggi M., Yallapu M.M., Chauhan S.C. (2020). Neutralization of SARS-CoV-2 Spike Protein via Natural Compounds: A Multilayered High Throughput Virtual Screening Approach. Curr. Pharm. Des..

[27] Yang X.J., Zhu H., Mu S.R., Wei W.J., Yuan X., Wang M., Liu Y., Hui J., Huang Y. (2019). Crystal structure of a Y-box binding protein 1 (YB-1)-RNA complex reveals key features and residues interacting with RNA. J. Biol. Chem..

[28] Erejuwa O.O., Sulaiman S.A., Ab Wahab M.S. (2013). Evidence in support of potential applications of lipid peroxidation products in cancer treatment. Oxidative Med. Cell. Longev..

[29] Click R.E. (2013). Anticancer activity and chemoprevention of xenobiotic organosulfurs in preclinical model systems. Oncol. Discov..

[30] Truffin D., Garçon G., Hannothiaux M.H., Colein P., Shirali P., Grave-Descampiaux B. (2003). Involvement of oxidative sress in the toxicity of 4-monochlorophenol in Hep G2 cells in culture. J. Appl. Toxicol..

[31] Coyne G.O., Kummar S., Meehan R.S., Do K., Collins J.M., Anderson L., Ishii K., Takebe N., Zlott J., Juwara L. (2020). Phase I trial of TRC102 (methoxyamine HCl) in combination with temozolomide in patients with relapsed solid tumors and lymphomas. Oncotarget.

[32] Pezuk J.A., Valera E.T., Delsin L.E., Scrideli C.A., Tone L.G., Brassesco M.S. (2015). The Antiproliferative and Pro-apoptotic Effects of Methoxyamine on Pediatric Medulloblastoma Cell Lines Exposed to Ionizing Radiation and Chemotherapy. Cent. Nerv. Syst. Agents Med. Chem..

[33] Saxena R., Yang C., Rao M., Turaga R.C., Garlapati C., Gundala S.R., Myers K., Ghareeb A., Bhattarai S., Kamalinia G. (2017). Preclinical Development of a Nontoxic Oral Formulation of Monoethanolamine, a Lipid Precursor, for Prostate Cancer Treatment. Clin. Cancer Res..

[34] Chiangjong W., Chutipongtanate S., Hongeng S. (2020). Anticancer peptide: Physicochemical property, functional aspect and trend in clinical application (Review). Int. J. Oncol..

[35] Maneikyte J., Bausys A., Leber B., Horvath A., Feldbacher N., Hoefler G., Strupas K., Stiegler P., Schemmer P. (2019). Dietary glycine decreases both tumor volume and vascularization in a combined colorectal liver metastasis and chemotherapy model. Int. J. Biol. Sci..

[36] Kvietkauskas M., Zitkute V., Leber B., Strupas K., Stiegler P., Schemmer P. (2021). Dietary Melatonin and Glycine Decrease Tumor Growth through Antiangiogenic Activity in Experimental Colorectal Liver Metastasis. Nutrients.

[37] Mikalauskas S., Mikalauskiene L., Bruns H., Nickkholgh A., Hoffmann K., Longerich T., Strupas K., Büchler M.W., Schemmer P. (2011). Dietary glycine protects from chemotherapy-induced hepatotoxicity. Amino Acids.

[38] Geeraerts S.L., Heylen E., De Keersmaecker K., Kampen K.R. (2021). The ins and outs of serine and glycine metabolism in cancer. Nat. Metab..

[39] Liu T., Xie X.L., Zhou X., Chen S.X., Wang Y.J., Shi L.P., Chen S.J., Wang Y.J., Wang S.L., Zhang J.N. (2021). Y-box binding protein 1 augments sorafenib resistance via the PI3K/Akt signaling pathway in hepatocellular carcinoma. World J. Gastroenterol..

