# Supplementary material for: Computational Repurposing and Experimental Validation of YBX1 Inhibitors in Hepatocellular Carcinoma
Source: Biomedicines. 2026 Feb 27;14(3):545. doi: 10.3390/biomedicines14030545 (PMC13023918; doi:10.3390/biomedicines14030545)
Supplement: Supplementary file 1 [file biomedicines-14-00545-s001.zip › biomedicines-4089997-supplementary.pdf]

Supplementary Figures

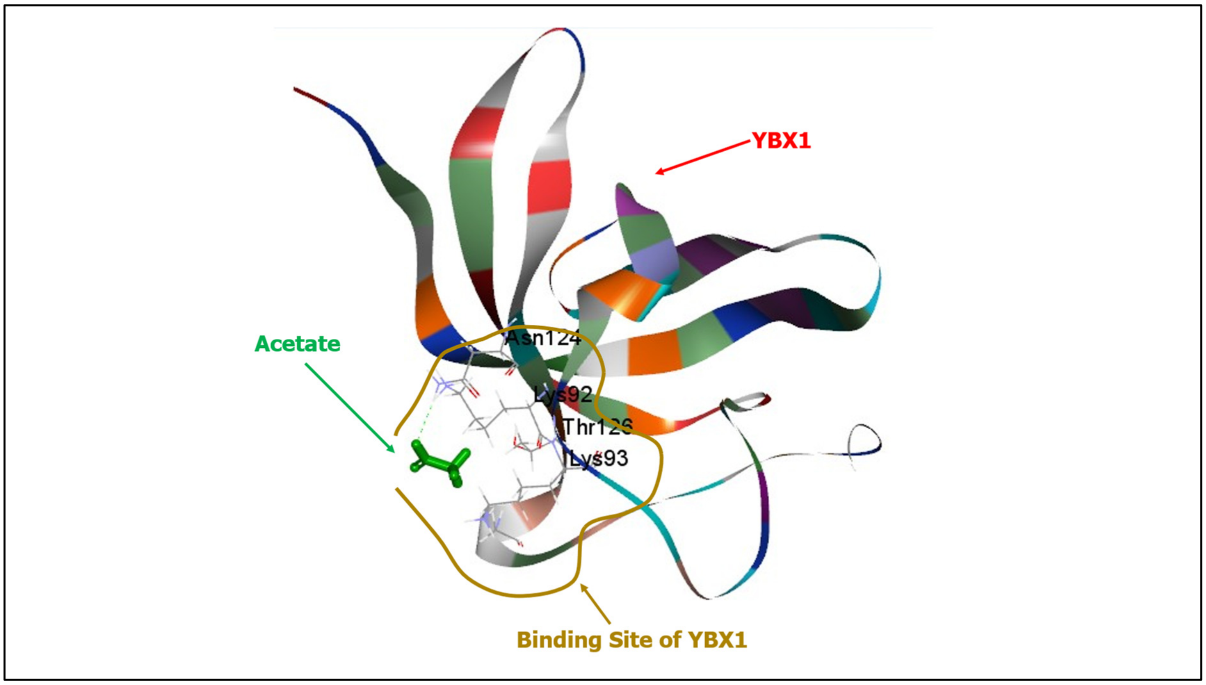

Figure S1: HTVS compound Acetate binding pattern analysis with YBX1. Binding pattern analysis image (generated by Discovery Studio Client) between the molecule Acetate (DrugBank ID: DB14511; PubChem ID: 175) and the YBX1 CSD (PDB ID: 6LMR).

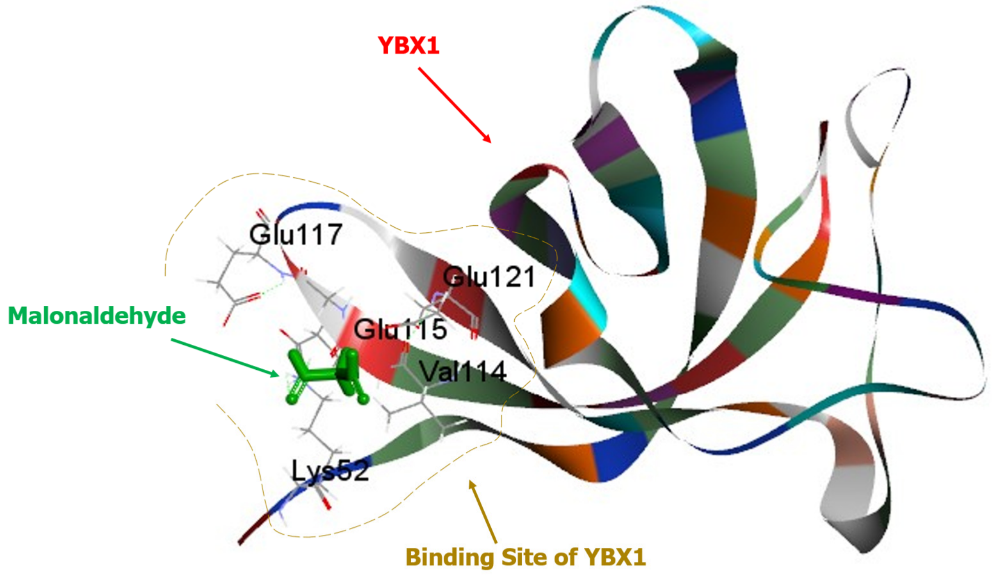

Figure S2: Malonaldehyde binding pattern analysis with YBX1. Binding pattern analysis image (generated by Discovery Studio Client) between the molecule Malonaldehyde (DrugBank ID: DB03057; PubChem ID: 10964) and the YBX1 CSD (PDB ID: 6LMR).

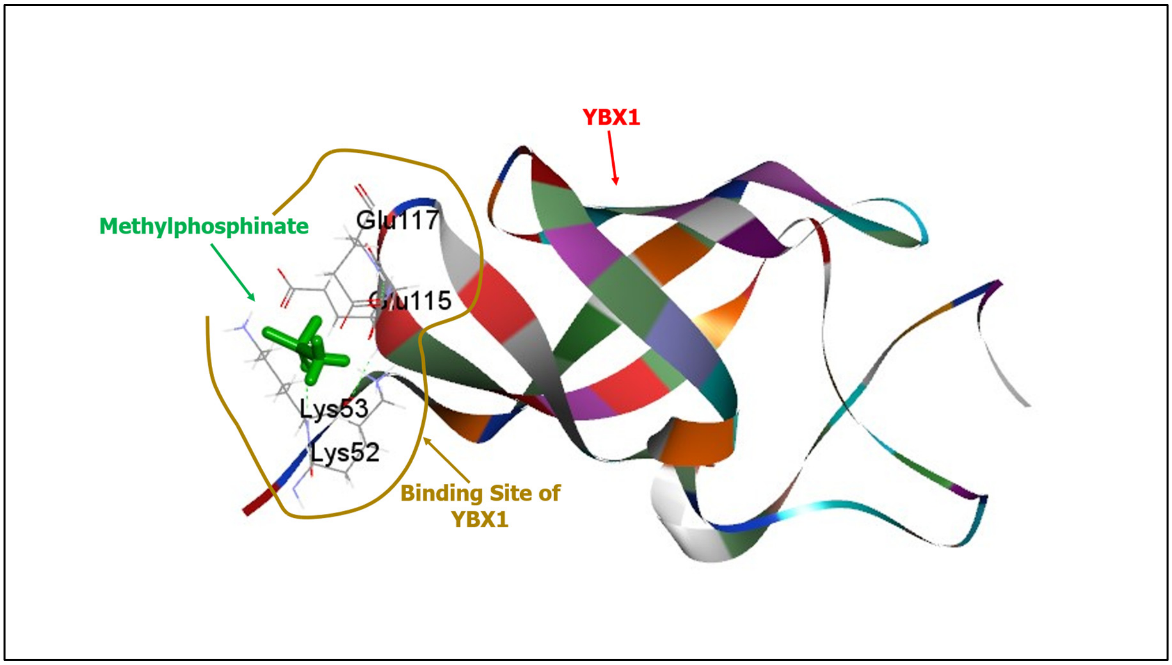

Figure S3: Methylphosphinate binding pattern analysis with YBX1. Binding pattern analysis image (generated by Discovery Studio Client) between the molecule Methylphosphinate (DrugBank ID: DB02825; PubChem ID: 3396559) and the YBX1 CSD (PDB ID: 6LMR).

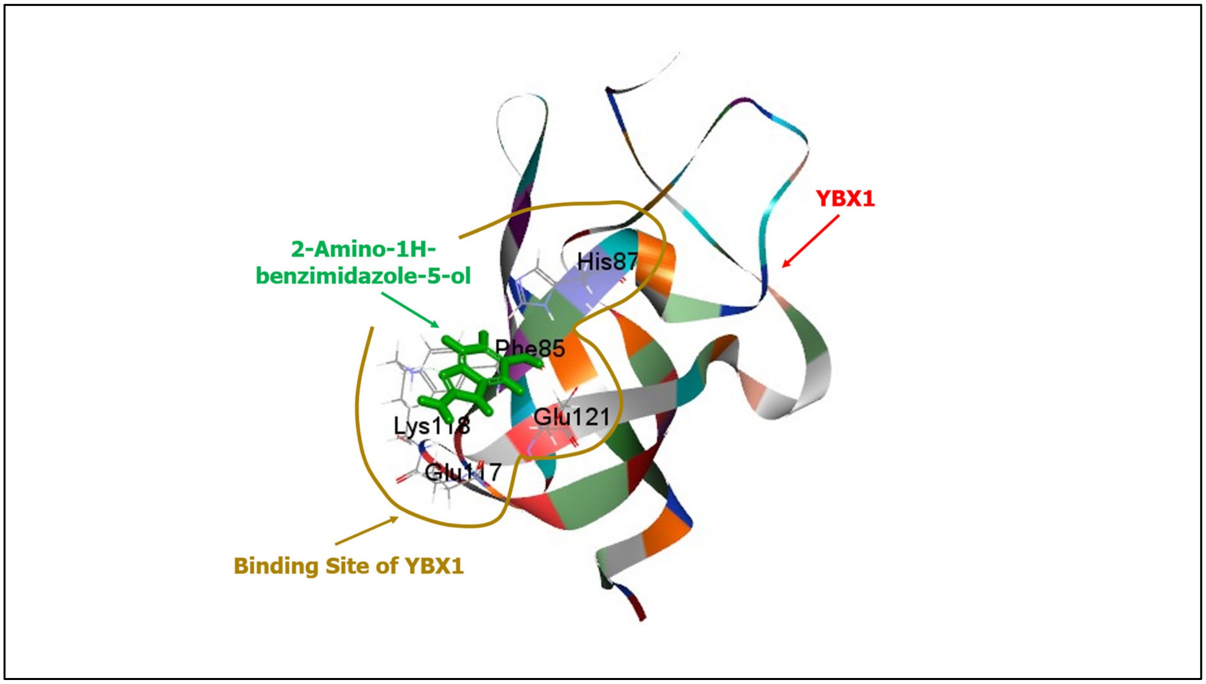

Figure S4: 2-Amino-1H-benzimidazole-5-ol binding pattern analysis with YBX1. Binding pattern analysis image (generated by Discovery Studio Client) between the molecule 2-amino-1H-benzimidazole-5-ol (DrugBank ID: DB03729; PubChem ID: 162636) and the YBX1 CSD (PDB ID: 6LMR).

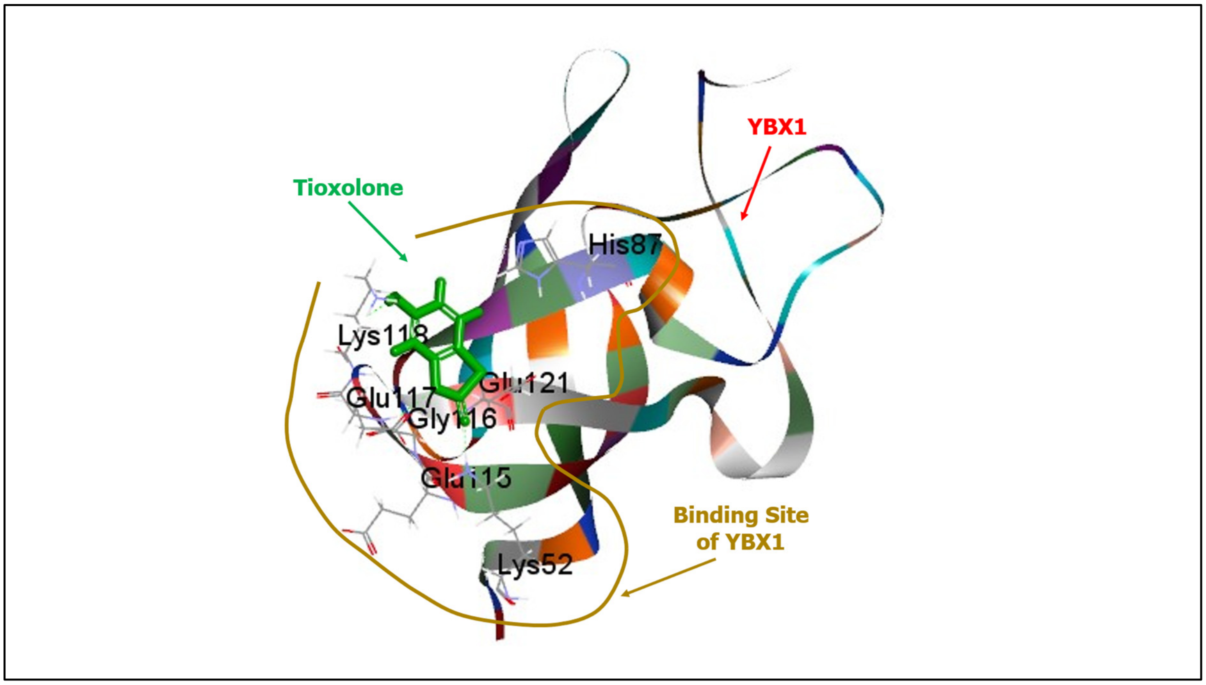

Figure S5: Tioxolone binding pattern analysis with YBX1. Binding pattern analysis image (generated by Discovery Studio Client) between the molecule Tioxolone (DrugBank ID: DB13343; PubChem ID: 72139) and the YBX1 CSD (PDB ID: 6LMR).

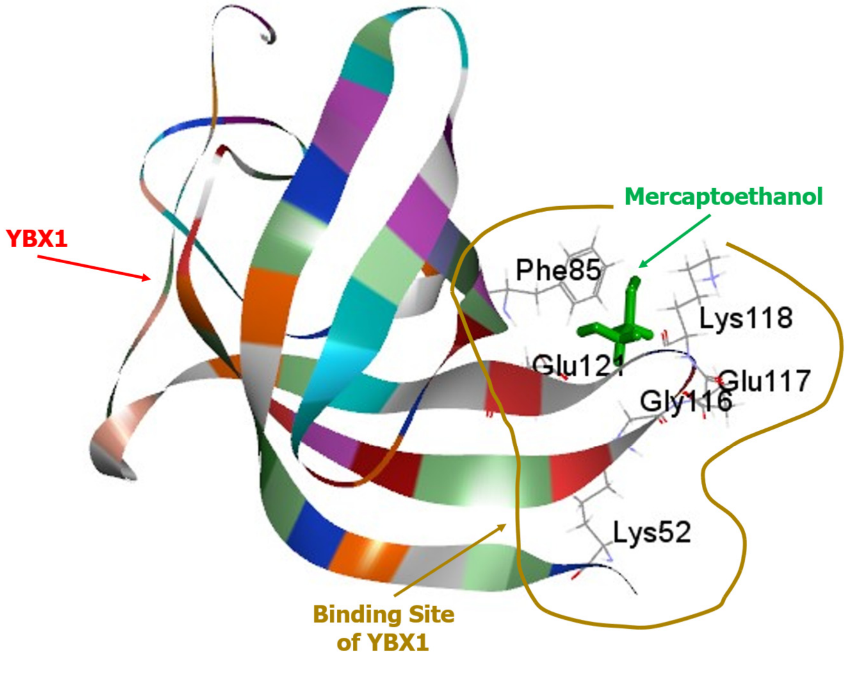

Figure S6: Mercaptoethanol binding pattern analysis with YBX1. Binding pattern analysis image (generated by Discovery Studio Client) between the molecule Mercaptoethanol (DrugBank ID: DB03345; PubChem ID: 1567) and the YBX1 CSD (PDB ID: 6LMR).

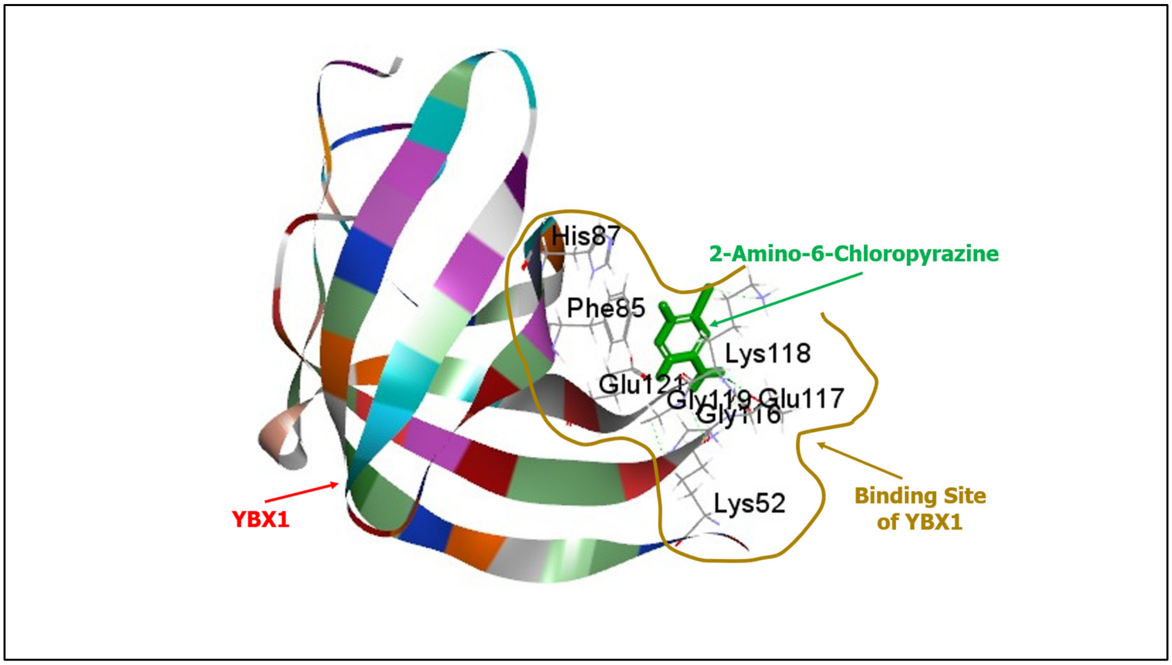

Figure S7: 2-Amino-6-Chloropyrazine binding pattern analysis with YBX1. Binding pattern analysis image (generated by Discovery Studio Client) between the molecule 2-Amino-6-Chloropyrazine (DrugBank ID: DB02297; PubChem ID: 118458) and the YBX1 CSD (PDB ID: 6LMR).

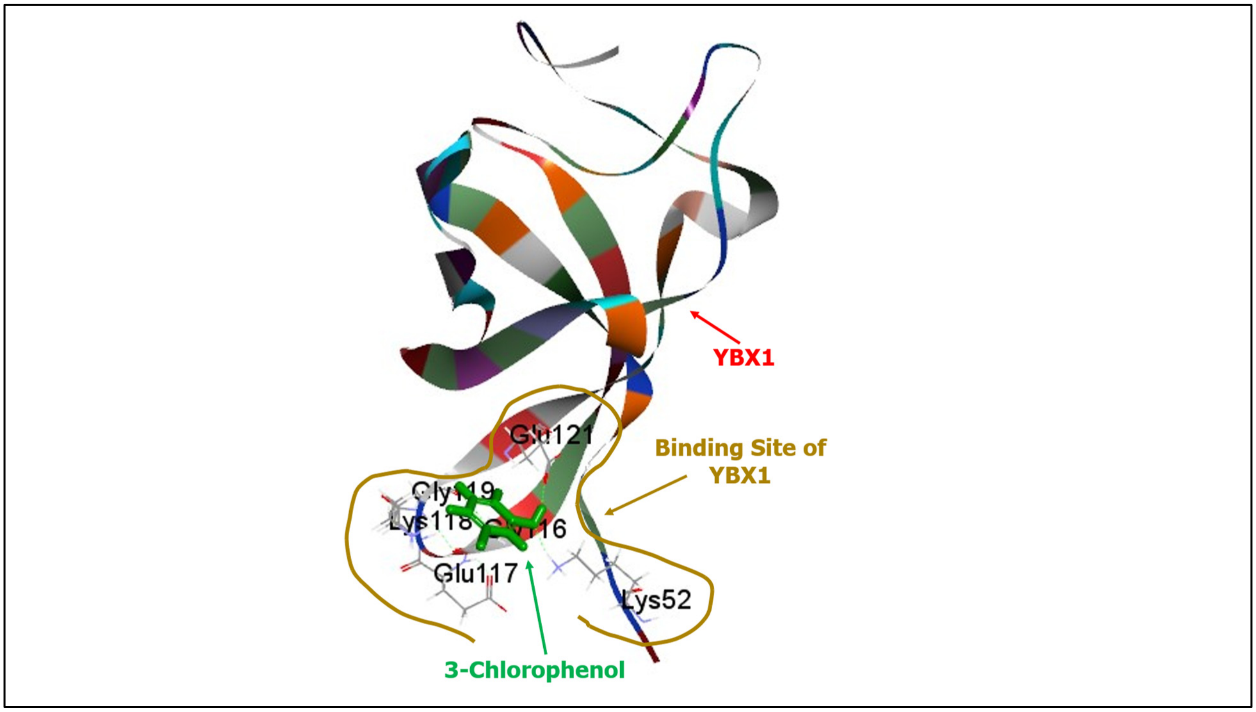

Figure S8: 3-Chlorophenol binding pattern analysis with YBX1. Binding pattern analysis image (generated by Discovery Studio Client) between the molecule 3-Chlorophenol (DrugBank ID: DB01957; PubChem ID: 7933) and the YBX1 CSD (PDB ID: 6LMR).

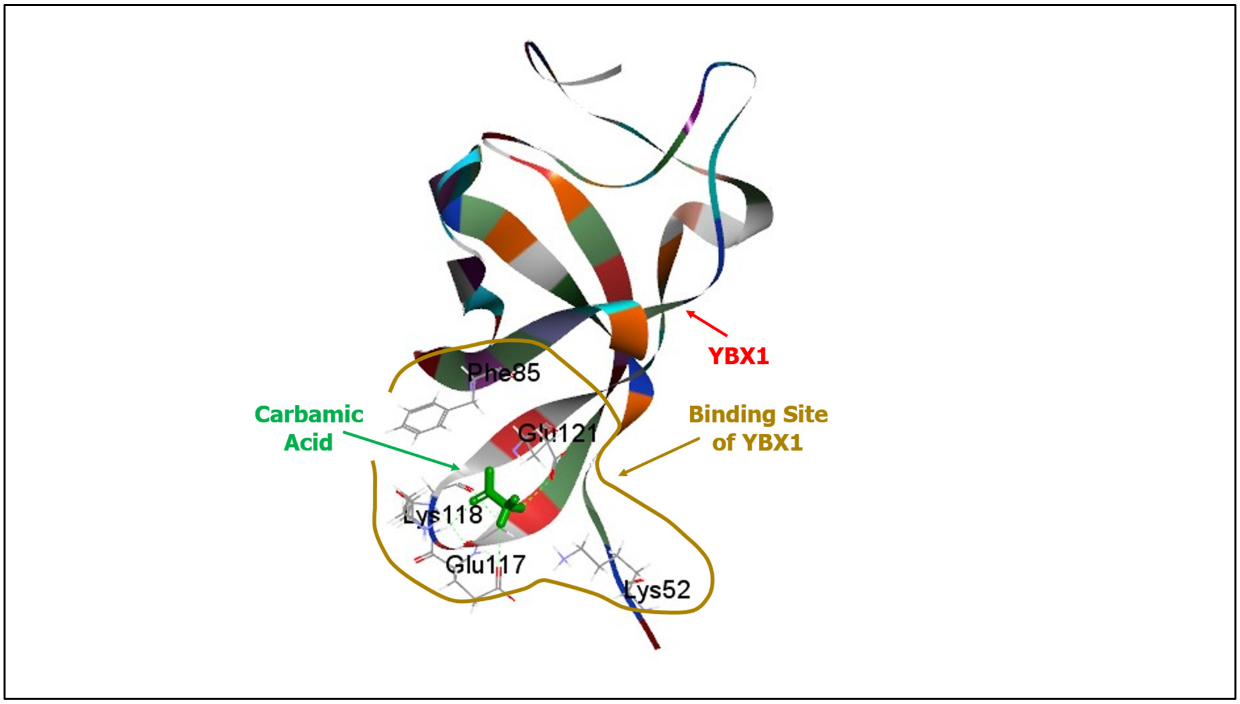

Figure S9: Carbamic Acid binding pattern analysis with YBX1. Binding pattern analysis image (generated by Discovery Studio Client) between the molecule Carbamic Acid (DrugBank ID: DB04261; PubChem ID: 57418154) and the YBX1 CSD (PDB ID: 6LMR).

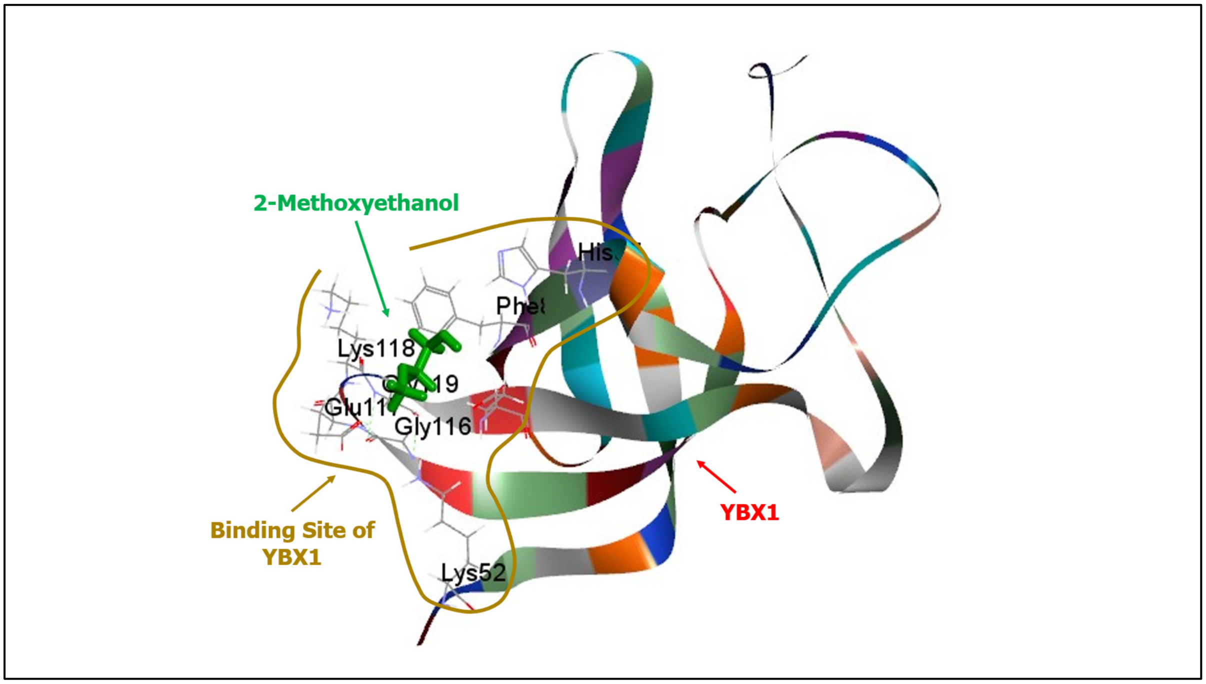

Figure S10: 2-Methoxyethanol binding pattern analysis with YBX1. Binding pattern analysis image (generated by Discovery Studio Client) between the molecule 2-Methoxyethanol (DrugBank ID: DB02806; PubChem ID: 8019) and the YBX1 CSD (PDB ID: 6LMR).

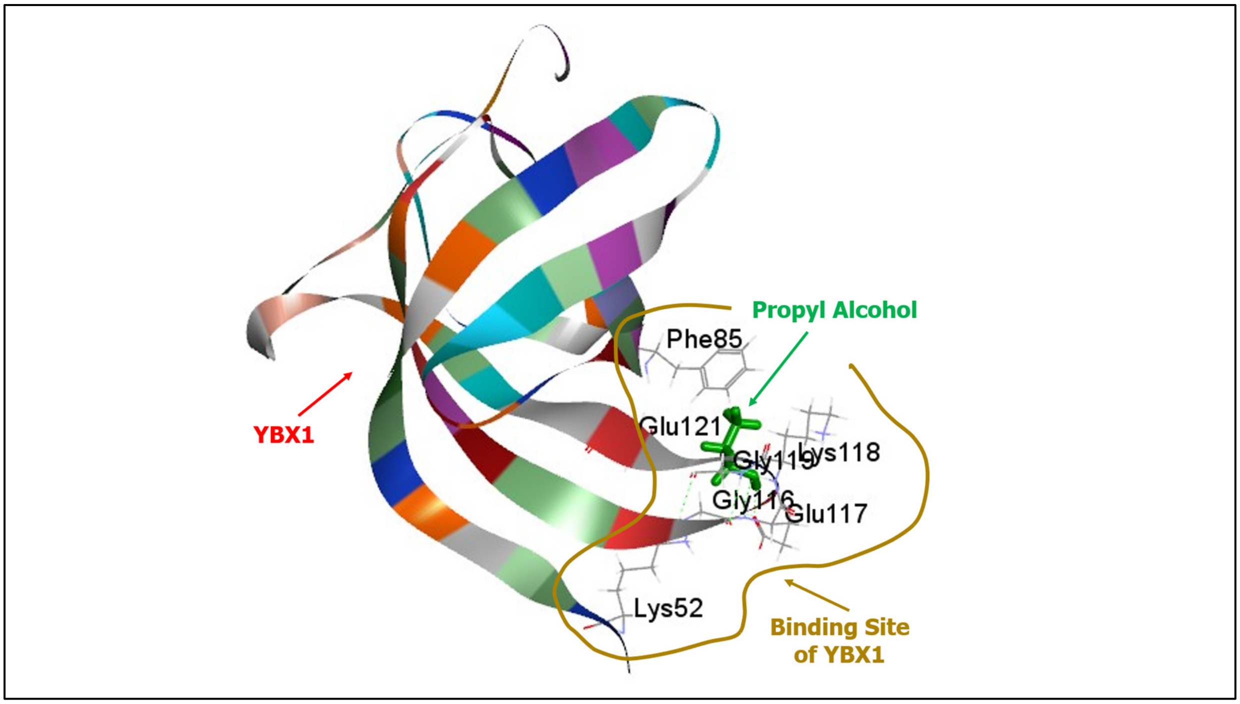

Figure S11: Propyl Alcohol binding pattern analysis with YBX1. Binding pattern analysis image (generated by Discovery Studio Client) between the Propyl Alcohol (DrugBank ID: DB03175; PubChem ID: 1031) and the YBX1 CSD (PDB ID: 6LMR).

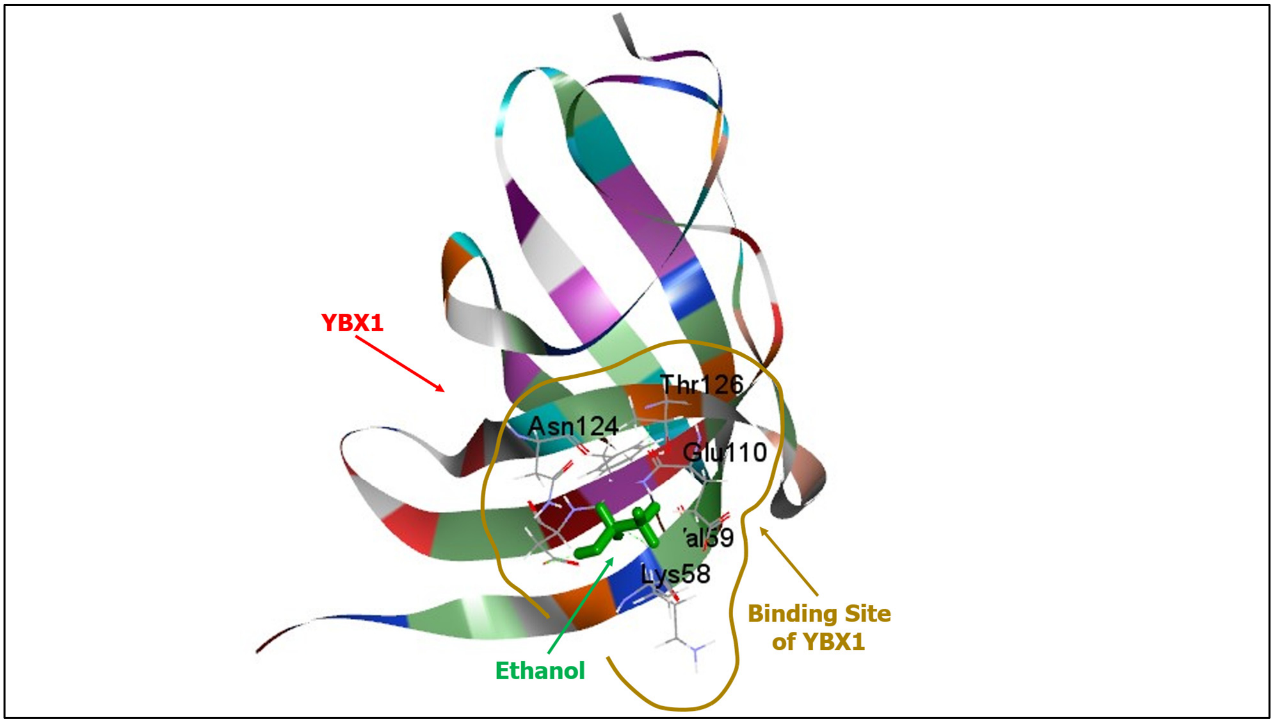

Figure S12: Ethanol binding pattern analysis with YBX1. Binding pattern analysis image (generated by Discovery Studio Client) between the Ethanol (DrugBank ID: DB00898; PubChem ID: 702) and the YBX1 CSD (PDB ID: 6LMR).

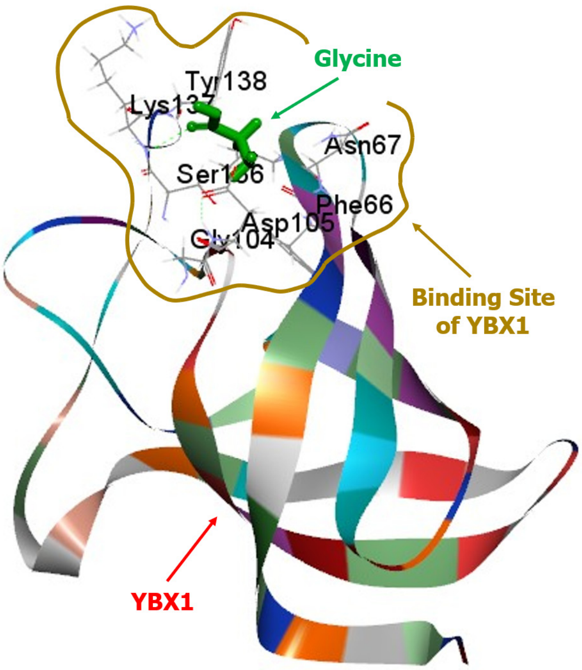

Figure S13: Glycine binding pattern analysis with YBX1. Binding pattern analysis image (generated by Discovery Studio Client) between the Glycine (DrugBank ID: DB00145; PubChem ID: 750) and the YBX1 CSD (PDB ID: 6LMR).

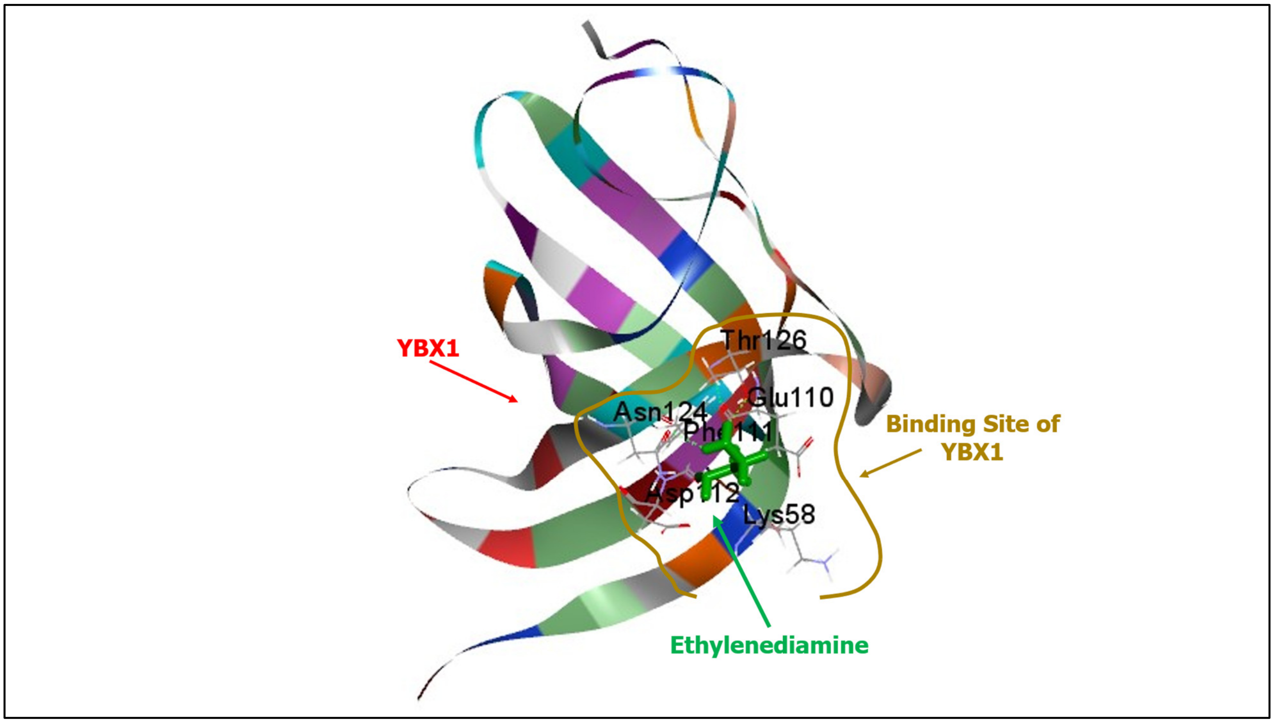

Figure S14: Ethylenediamine binding pattern analysis with YBX1. Binding pattern analysis image (generated by Discovery Studio Client) between the Ethylenediamine (DrugBank ID: DB14189; PubChem ID: 3301) and the YBX1 CSD (PDB ID: 6LMR).

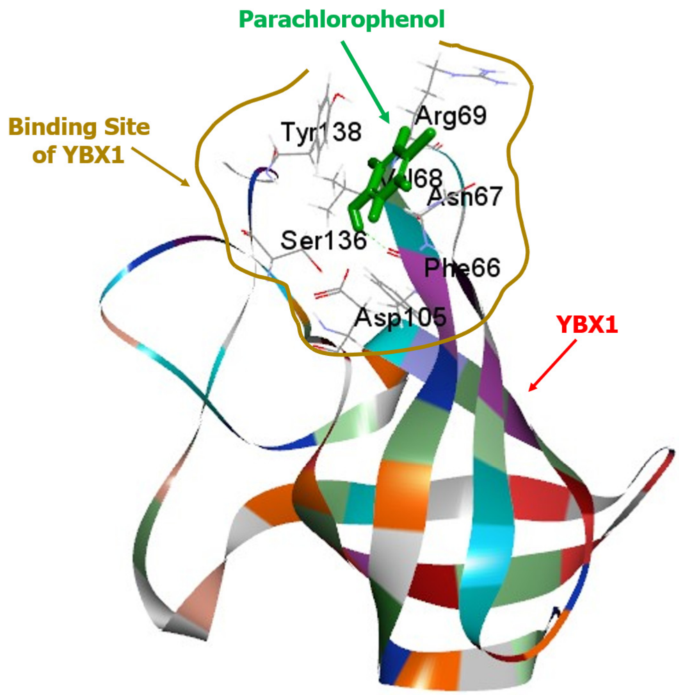

Figure S15: Parachlorophenol binding pattern analysis with YBX1. Binding pattern analysis image (generated by Discovery Studio Client) between the Parachlorophenol (DrugBank ID: DB13154; PubChem ID: 4684) and the YBX1 CSD (PDB ID: 6LMR).

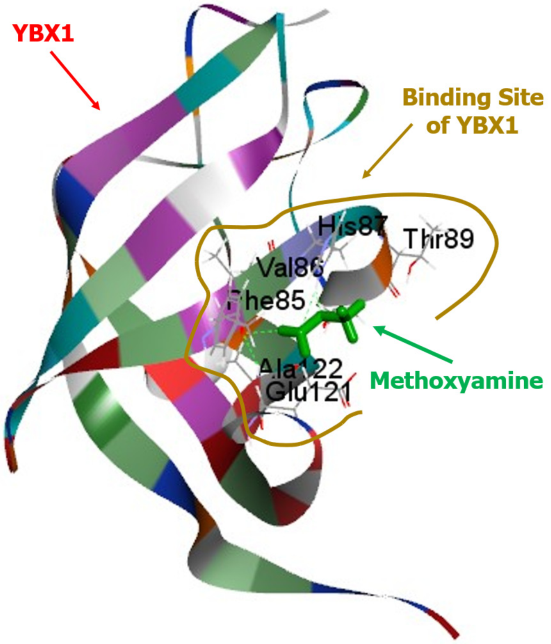

Figure S16: Methoxyamine binding pattern analysis with YBX1. Binding pattern analysis image (generated by Discovery Studio Client) between the Methoxyamine (DrugBank ID: DB06328; PubChem ID: 4113) and the YBX1 CSD (PDB ID: 6LMR).

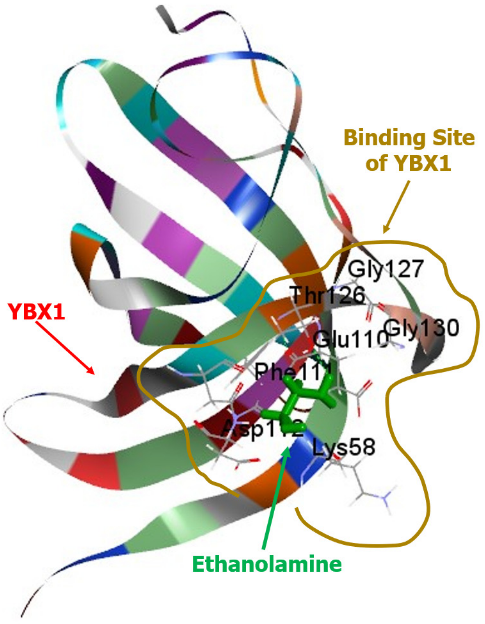

Figure S17: Ethanolamine binding pattern analysis with YBX1. Binding pattern analysis image (generated by Discovery Studio Client) between the Ethanolamine (DrugBank ID: DB03994; PubChem ID: 700) and the YBX1 CSD (PDB ID: 6LMR).

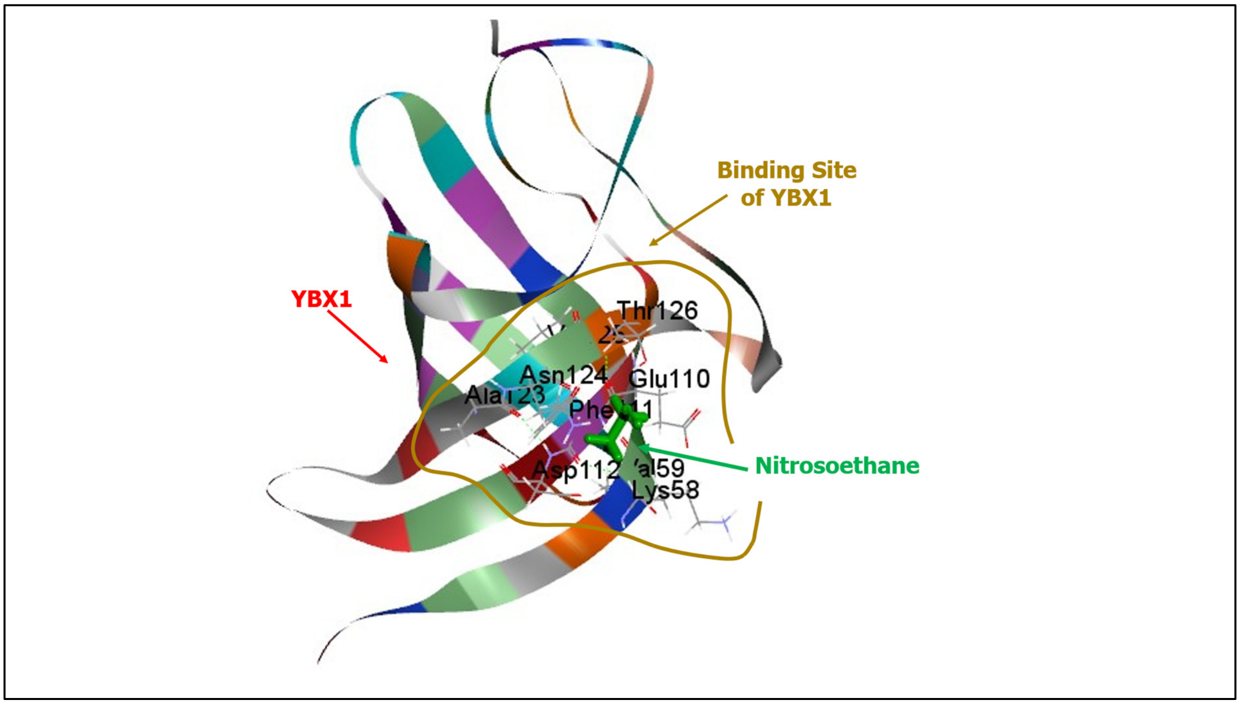

Figure S18: Nitrosoethane binding pattern analysis with YBX1. Binding pattern analysis image (generated by Discovery Studio Client) between the Nitrosoethane (DrugBank ID: DB02646; PubChem ID: 79124) and the YBX1 CSD (PDB ID: 6LMR).

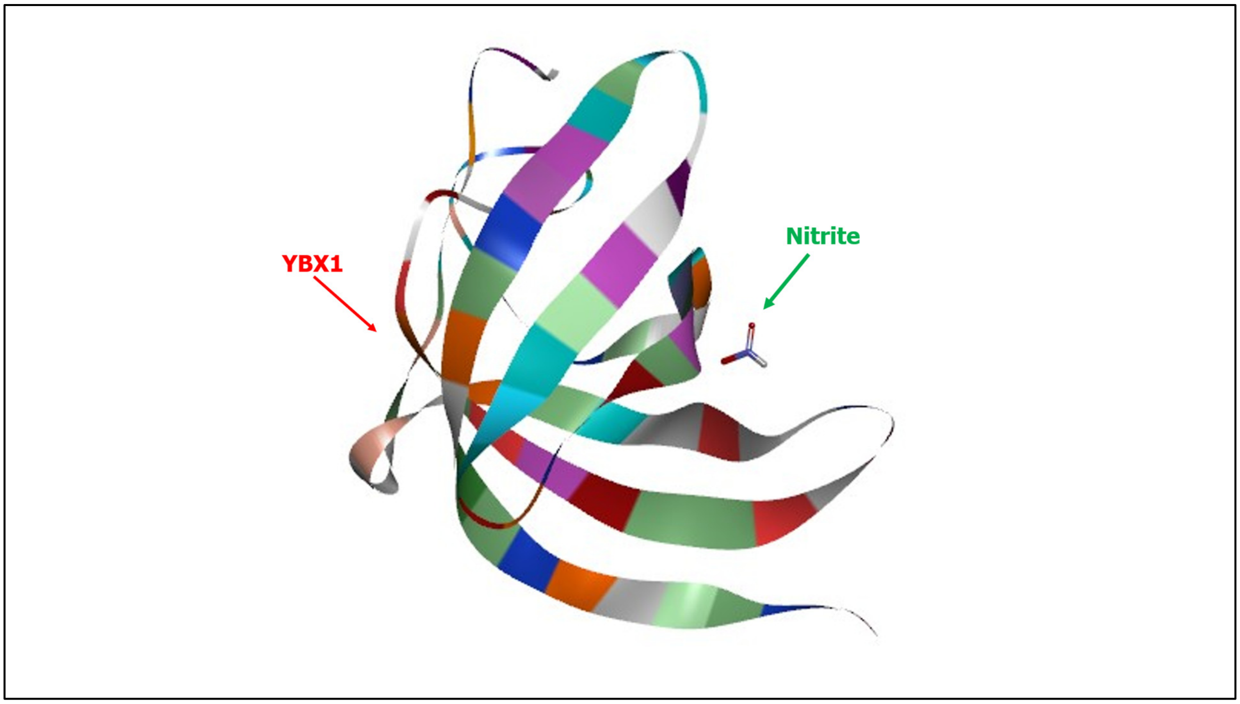

Figure S19: Nitrite binding pattern analysis with YBX1. Binding pattern analysis image (generated by Discovery Studio Client) between the Nitrite (DrugBank ID: DB12529; PubChem ID: 946) and the YBX1 CSD (PDB ID: 6LMR).

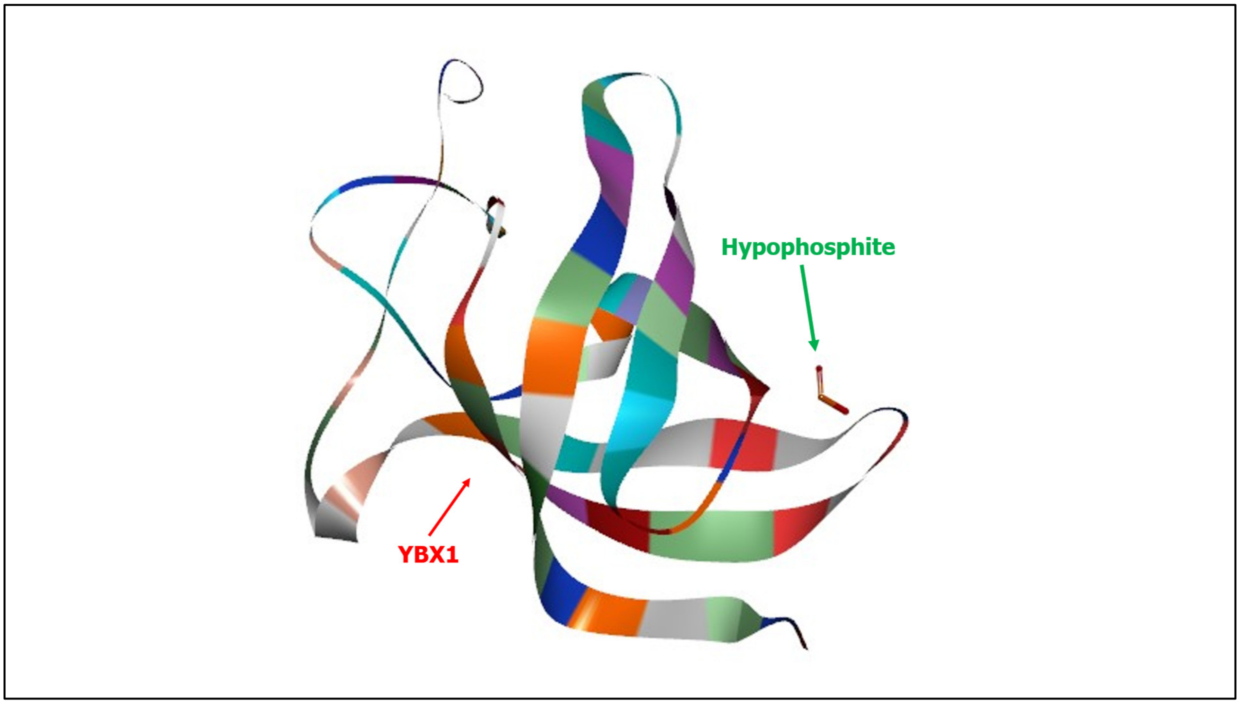

Figure S20: Hypophosphite binding pattern analysis with YBX1. Binding pattern analysis image (generated by Discovery Studio Client) between the Hypophosphite (DrugBank ID: DB04053; PubChem ID: 183145) and the YBX1 CSD (PDB ID: 6LMR).

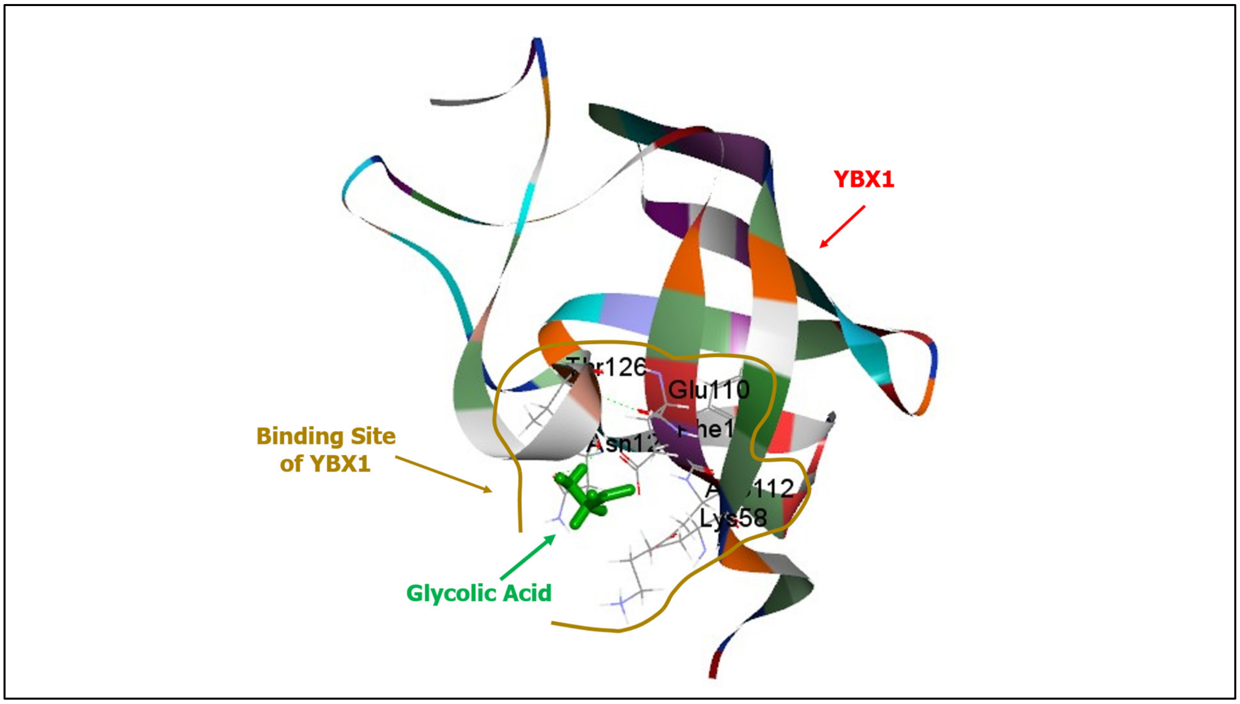

Figure S21: Glycolic Acid binding pattern analysis with YBX1. Binding pattern analysis image (generated by Discovery Studio Client) between the Glycolic Acid (DrugBank ID: DB03085; PubChem ID: 3698251) and the YBX1 CSD (PDB ID: 6LMR).

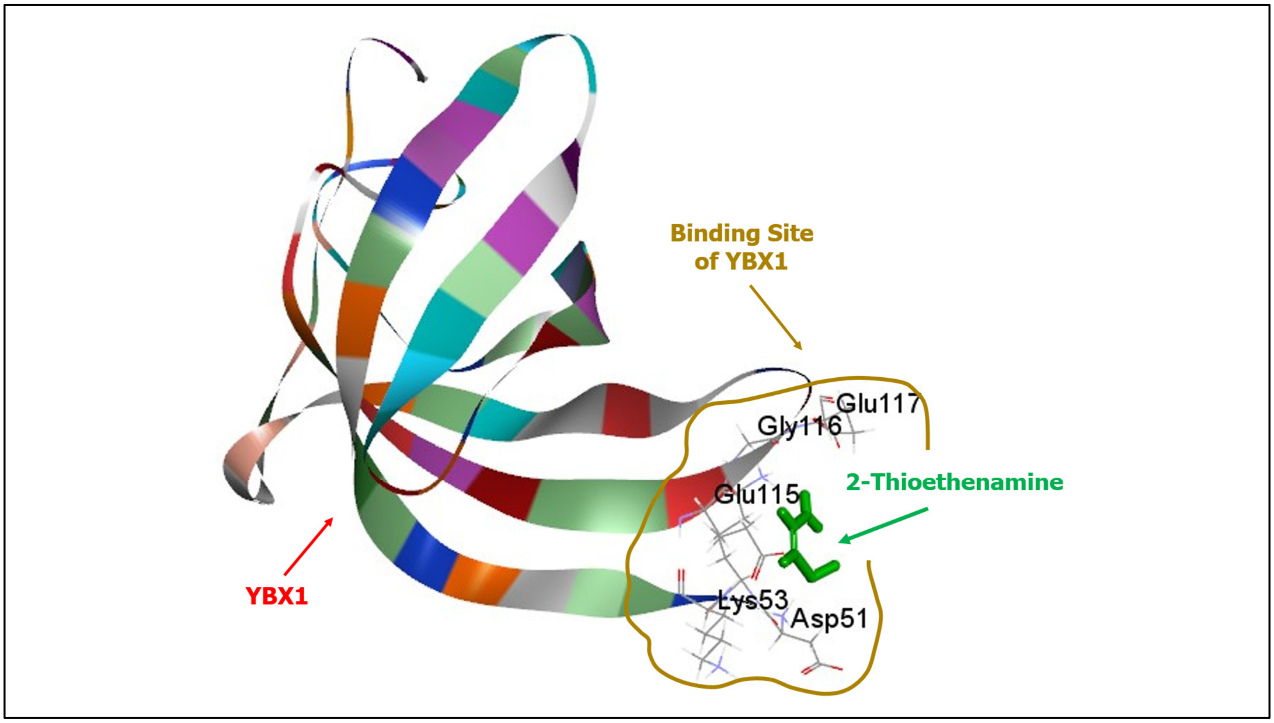

Figure S22: 2-Thioethenamine binding pattern analysis with YBX1. Binding pattern analysis image (generated by Discovery Studio Client) between the 2-Thioethenamine (DrugBank ID: DB01968; PubChem ID: 17754199) and the YBX1 CSD (PDB ID: 6LMR).

Supplementary Tables

Supplementary Table S1: Details of antibodies used in the study

YBX1	Abcam	ab15580
actin	Merk	A5441

Supplementary Table S2: RT-PCR Primers used in this study

Beta actin F	5′-GCA TGG GTC AGA AGG ATT CC-3′
Beta actin R	5′-AGG ATG CTG CTC TTG CTC TG-3′
YBX1 F	5′-CCC CAG GAA GTA CCT TCG C-3′
YBX1 R	5′- AGC GTC TAT AAT GGT TAC GGT CT-3′
MDR1 F	5′- GGG AGCC TTA ACA CCC GAC TTA 3′
MDR1 R	5′- GCC AAA ATC ACA AGG GTT AGC TT-3′
VEGF F	5′ -GGC TGG CAA CAT AAC AGA GAA -3′
VEGF R	5′- CCC CAC ATC TAT ACA CAC CTC C-3′
